# Assessing a biomarker’s ability to reduce invasive procedures in patients with benign lung nodules: Results from the ORACLE study

**DOI:** 10.1371/journal.pone.0287409

**Published:** 2023-07-11

**Authors:** Michael A. Pritchett, Barry Sigal, Mark R. Bowling, Jonathan S. Kurman, Trevor Pitcher, Steven C. Springmeyer, for the ORACLE Study Investigators

**Affiliations:** 1 Department of Pulmonary Medicine, FirstHealth of the Carolinas & Pinehurst Medical Clinic, Pinehurst, North Carolina, United States of America; 2 Southeastern Research Center, Winston-Salem, North Carolina, United States of America; 3 Division of Pulmonary, Critical Care, and Sleep Medicine, Brody School of Medicine, Eastern Carolina University, Greenville, North Carolina, United States of America; 4 Division of Critical Care Medicine, Interventional Pulmonology, Pulmonary Disease, Medical College of Wisconsin, Milwaukee, Wisconsin, United States of America; 5 Medical Affairs, Biodesix, Inc., Boulder, Colorado, United States of America; Taichung Veterans General Hospital, TAIWAN

## Abstract

A blood-based integrated classifier (IC) has been clinically validated to improve accuracy in assessing probability of cancer risk (pCA) for pulmonary nodules (PN). This study evaluated the clinical utility of this biomarker for its ability to reduce invasive procedures in patients with pre-test pCA ≤ 50%. This was a propensity score matching (PSM) cohort study comparing patients in the ORACLE prospective, multicenter, observational registry to control patients treated with usual care. This study enrolled patients meeting the intended use criteria for IC testing: pCA ≤ 50%, age ≥40 years, nodule diameter 8–30 mm, and no history of lung cancer and/or active cancer (except for non-melanomatous skin cancer) within 5 years. The primary aim of this study was to evaluate invasive procedure use on benign PNs of registry patients as compared to control patients. A total of 280 IC tested, and 278 control patients met eligibility and analysis criteria and 197 were in each group after PSM (IC and control groups). Patients in the IC group were 74% less likely to undergo an invasive procedure as compared to the control group (absolute difference 14%, p <0.001) indicating that for every 7 patients tested, one unnecessary invasive procedure was avoided. Invasive procedure reduction corresponded to a reduction in risk classification, with 71 patients (36%) in the IC group classified as low risk (pCA < 5%). The proportion of IC group patients with malignant PNs sent to surveillance were not statistically different than the control group, 7.5% vs 3.5% for the IC vs. control groups, respectively (absolute difference 3.91%, p 0.075). The IC for patients with a newly discovered PN has demonstrated valuable clinical utility in a real-world setting. Use of this biomarker can change physicians’ practice and reduce invasive procedures in patients with benign pulmonary nodules.

**Trial registration**: Clinical trial registration: ClinicalTrials.gov NCT03766958.

## Introduction

With the increasing use of chest computed tomography (CT) for a myriad of clinical indications, pulmonary nodules (PNs) have become an increasingly common clinical problem encountered by clinicians. By current estimates, over 1.5 million lung nodules are detected in the United States (US) annually and the evaluation of nodules represents a significant burden to the healthcare system [1].

Management of patients with PNs is determined by physician judgment, often guided by a quantitative risk model, such as the Mayo Solitary Pulmonary Nodule (SPN) calculator, coupled with threshold-based decision making and evidence based clinical guidelines [2–5]. To assess the likelihood of malignancy, a calculator commonly incorporates patient characteristics (e.g., age, smoking history, and personal history of cancers) with radiological characteristics (e.g., nodule diameter, lobe location, and presence or absence of spiculation). Based upon American College of Chest Physician (ACCP) guidelines, patients with a pre-test probability of cancer (pCA) < 5% should undergo active surveillance, those ≥ 5% to 65% are recommended to have further testing (e.g., Positron Emission Tomography (PET) or biopsy (bronchoscopy or transthoracic needle biopsy [TTNB]) and those with pCA ≥ 65% should be considered for a bronchoscopy, TTNB or surgery [2]. However, quantitative risk models are not always accurate and researchers have attempted to find other minimally invasive ways to aid risk stratification, improve clinical practice and patient outcomes [6, 7].

Blood biomarkers have been a focus of study in the past decade with the goal of differentiating benign from malignant nodules. A proteomic integrated classifier (Nodify XL2®, (IC), Biodesix Inc., Boulder CO.) was developed to help physicians to identify nodules that are likely benign and may benefit from active surveillance [8]. A prospective, multicenter observational trial (PANOPTIC, NCT01752114) of patients with indeterminate pulmonary nodules with a pre-test pCA ≤ 50% (8–30 mm in largest diameter) demonstrated the blood-based IC’s sensitivity to be 97% and the negative predictive value to be 98%, indicating that tested nodules are characterized as likely benign [9].

We undertook this study using a propensity score matched (PSM) cohort study design to assess the impact of the IC on guiding physician decisions to reduce unnecessary invasive procedures in patients with low to intermediate risk PN’s with a probability of cancer of ≤ 50%.

## Material and methods

### Study design and objectives

The prospective ORACLE observational research registry (October 2018 to March 2020) is a multicenter cohort assembled to evaluate the impact of an IC on physician decision-making when used in the clinical management of a recently identified PNs with Mayo SPN pCA ≤ 50% as compared to a historical control population from the same institutions. A total of 15 community and academic pulmonary practices covering a wide geographic area participated in the study and the institutional review boards at each site or centrally approved the study. Written consent was obtained from all eligible patients and patient follow-up was continued until all eligible patients had completed at least one year.

The primary objective of the ORACLE study was to show a statistically significant reduction in the proportion of benign PNs experiencing invasive procedures (biopsies or surgery) between a prospective group of patients managed by the IC test and a control group managed by usual care. Additionally, the secondary objective of the study was to show a statistically significant noninferiority of the proportion of surveilled PNs resulting in a malignant diagnosis, under management with the IC test as compared to a control group managed by usual care.

The sample size for the ORACLE study was powered based on the secondary objective due to larger patient numbers required over the primary objective. A power analysis indicated that with a noninferiority margin of 10% relative to management without the IC (control group), 453 patients per group would be necessary to reject the null hypothesis with 80% power and type 1 error α = 0.05, one-sided.

### Patient selection

Study inclusion criteria were pre-test pCA ≤ 50%, ≥ 40 years of age, nodule 8–30 mm in diameter, and no history of lung cancer and/or active cancer (except for non-melanomatous skin cancer) within 5 years. Study inclusion criteria were chosen to mirror the intended use population defined in the clinical validation study PANOPTIC, specifically patients presenting with an indeterminant PN with low to moderate probability of malignancy (pCA ≤ 50%) [9]. Patients with multiple nodules were not excluded, but an identified nodule of concern (generally the largest nodule) was required. Patients were excluded if a biopsy had been attempted or completed after the first CT scan identifying the nodule of concern but before enrollment or if patients were deemed high cancer risk (pCA > 50%) by physician assessment. Further exclusion criteria included concurrent participation in any unrelated clinical trial that could alter the management of the patient’s nodule of concern, or any illness or factor that would prevent compliance with follow-up testing. Benign diagnosis was determined by specific benign histopathology (e.g., granulomatous inflammation, yeast, etc.) or sequential CT imaging (nodule resolved or stable for at least 1 year).

### Control population

To establish a control group that was comparable to the ORACLE registry patients, a cohort of patients were enrolled by chart review at the investigative sites. Chart review patients had a nodule identified between June 2015 and September 2018, prior to the initiation of the prospective ORACLE study (commenced October 2018). Per protocol, retrospective chart review subjects were identified systematically (random selection or consecutively) to reduce selection bias. The inclusion criteria for the chart review patients were pre-test pCA ≤ 50%, ≥ 40 years of age, nodule 8–30 mm in diameter, and no history of lung cancer and/or active cancer (except for non-melanomatous skin cancer) within 5 years. Consistent with the prospective population, the control patient diagnoses were determined either by histopathology or sequential CT imaging (nodule resolved or stable for at least 1 year). The data collection procedures, inclusion, and exclusion criteria were designed to match the intended use criteria for the test and to be identical to the prospective registry patients ensuring comparability between the two groups.

### Data collection

At enrollment, the pre-test pCA was calculated and patient baseline demographic and nodule characteristics were collected. Blood was drawn at enrollment prior to any further testing or procedures. Liquid chromatography mass spectrometry (multiple reaction monitoring) analysis of blood samples was performed in a centralized Clinical Laboratory Improvement Amendments (CLIA) certified, College of American Pathologists (CAP) accredited, New York State Clinical Laboratory Evaluation Program (CLEP) approved, and ISO 13485:2016 certified clinical laboratory (Biodesix, Inc., De Soto, Kansas laboratory). Biomarker results were reported to physicians in as short as 6 days and post-test treatment courses were recorded. Registry data were collected at enrollment and at least every 6 months for up to 24 months or until an endpoint (such as definitive diagnosis, or nodule resolution on follow-up CT scan) was met. The study protocol did not mandate specific follow up recommendations, and nodule management was according to clinical judgment in combination with the IC test results. Data were extracted from procedure reports, test results, physical exams or historical medical records and entered by site investigators into electronic case report forms housed on a secure, access-controlled, validated, web-based electronic data capture system. Routine monitoring visits were conducted onsite (or remotely if required).

### Blood-based integrated classifier

The IC (Nodify XL2®, Biodesix Inc. Boulder, CO) test comprises measurement of two plasma proteins, Galectin-3 Binding Protein (LG3BP) and Scavenger Receptor Cysteine-Rich Protein Type 1 Protein M130 (C163A), by liquid chromatography-multiple reaction monitoring (MRM) mass spectroscopy and integration with five clinical risk factors in an algorithm to determine a post-test probability result of likely benign with a negative predictive value (NPV) of 98%, as previously described [8–10]. The clinical risk factors are age (in years), smoking status (never, current/former), nodule diameter (largest diameter in mm), edge characteristics (spiculated or not), and location (upper lobe or not).

### Statistical analysis

Considering that the two patient groups (IC Tested & Control) were not randomized, Propensity Score Matching (PSM) was performed to balance the distributions of baseline covariates and cancer prevalence between the two groups [11, 12]. Seven matching variables likely to impact the decision to undergo an invasive procedure were identified and used for matching in accordance with recommendations in the literature and expert opinion [13, 14]. The variables were: nodule size (in mm), spiculation (present or absent), nodule location (upper, or other lobe), nodule type (solid or non-solid), age (years), smoking status (current/ former, never), and sex. In addition, final diagnosis (benign or cancer) was used as a matching variable to balance cancer prevalence. Logistic regression was applied across the 8 variables to calculate a propensity score used in constructing matched pairs between the IC tested and untested groups. Patients in the IC Tested and Control groups were matched 1:1 without replacement using the nearest neighbor matching method. A caliper of 0.2 was chosen to optimally reduce the paired differences between the propensity scores of the IC Tested and Control groups, while maximizing the number of patients matched.

Statistical analyses were carried out using R (version 4.0.4 or later, The R Project, https://www.r-project.org/) and plots were produced using R and PRISM 8 (GraphPad, La Jolla, CA). Categorical variables were compared using the Chi-squared or Fisher Exact tests, continuous variables were compared using Student’s T test; differences in proportions (absolute and relative) and odds ratios were evaluated using the Wald Test (Z test). A p <0.05 was considered significant. P values and 95% confidence intervals (CI) for the PSM population were adjusted for cluster effects using cluster robust standard error adjusted CIs and p values [15].

## Results

**Fig 1:**
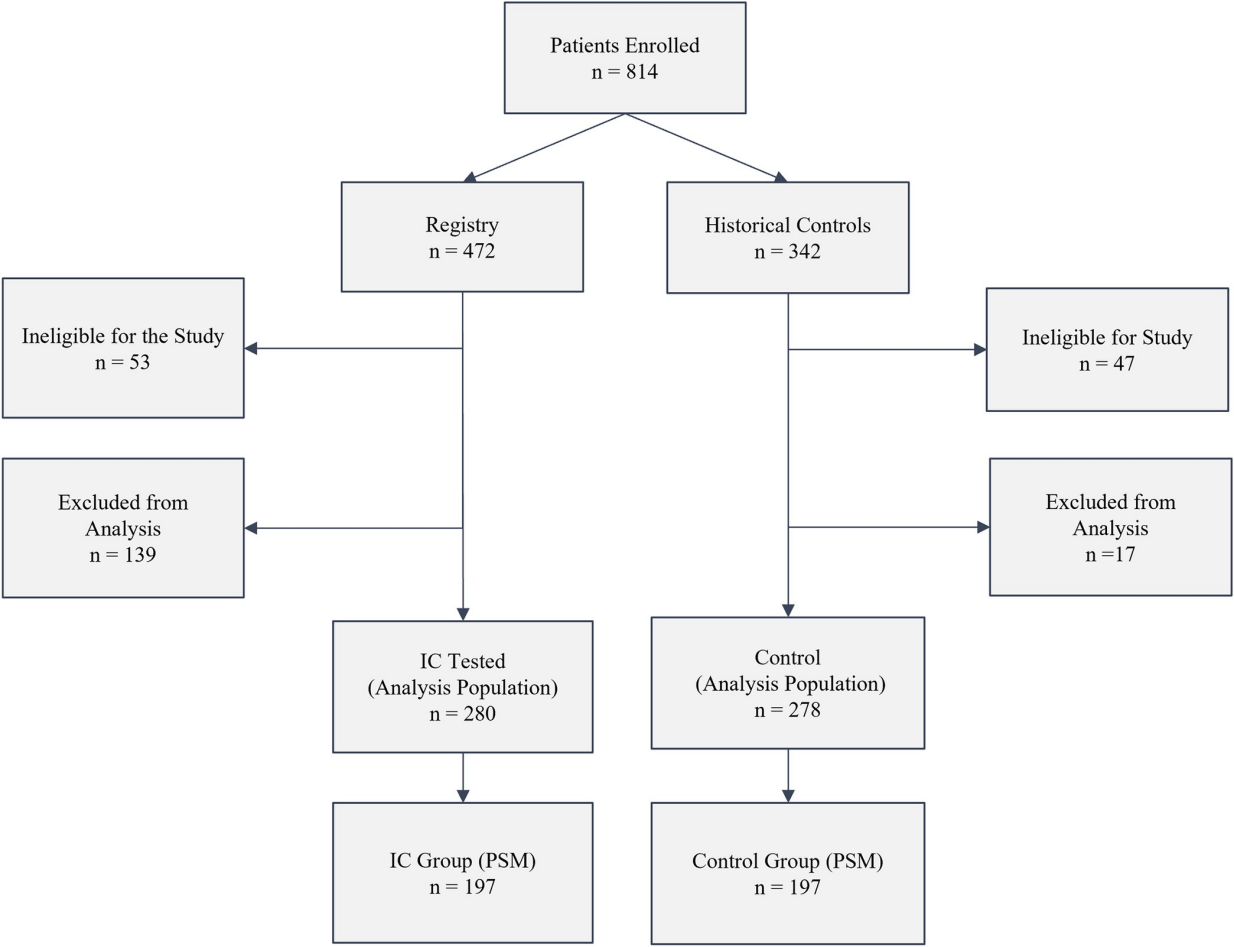
Patient enrollment in ORACLE and eligibility for analysis. All IC tested patients in the eligible population could be analyzed for reclassification by the integrated classifier. Registry patients were determined to be ineligible (n = 53) for the following reasons: pre-test pCA > 50% (n = 23), patients not meeting the study eligibility criteria (n = 13), history of non-lung cancer within 5 years of study enrollment (n = 5), previous history of lung cancer (n = 4), baseline CT scan over 90 days prior to enrollment (n = 3), no baseline CT scan (n = 2), < 40 years of age (n = 2), no blood sample submitted (n = 1). Registry patients were excluded from the analysis population (n = 139) for the following reasons: procedure performed prior to receipt of test result (n = 51), lost to follow (n = 37), incomplete clinical data (n = 24), patient deceased prior to endpoint (n = 8), presumptive treatment (e.g. SBRT) without diagnosis (n = 6), withdrew consent (n = 5), tumor other than NSCLC or SCLC (n = 4), other (n = 3), and nodule diagnosis prior to study initiation (n = 1). Control patients were deemed ineligible for the following reasons (n = 47): pCA >50% (n = 15), did not meet eligibility criteria (n = 18), and no diagnosis (n = 14). Controls were excluded from the analysis (n = 17) for the following reasons: presumptive treatment (n = 10), tumor other than NSCLC or SCLC (n = 5) and incomplete clinical data (n = 2).

**Fig 2:**
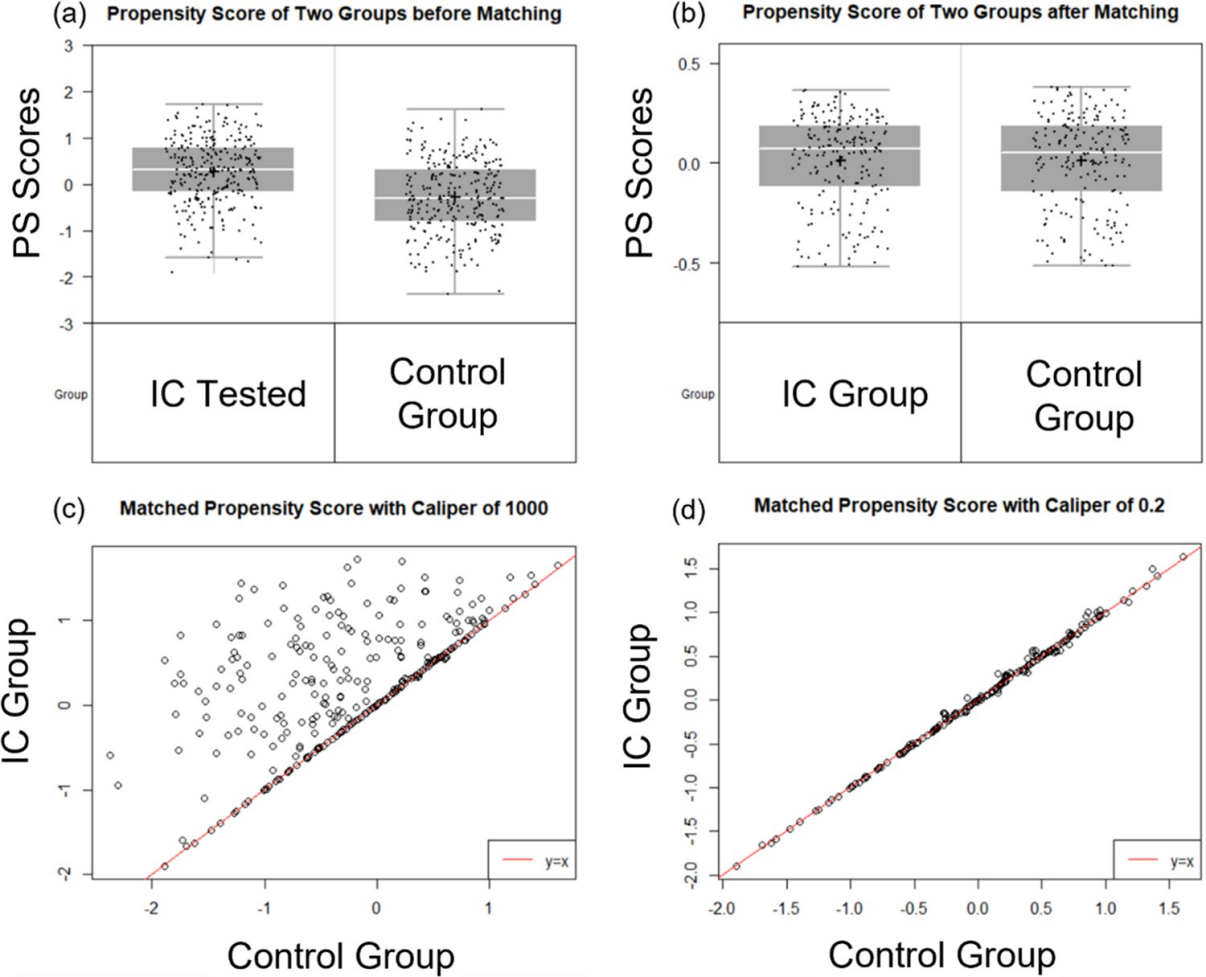
Propensity score matching metrics. Panel A shows the distribution of propensity scores for the IC tested (mean score 0.27(SD = 0.71)) and the control group (mean score -0.28 (SD = 0.77)) prior to propensity score matching, indicating a significant difference in propensity scores (p <0.001). Panel B shows the distribution of propensity scores following nearest neighbor matching (1:1, without replacement), with the mean score for the IC group of 0.03 (SD = 0.66) and control group mean score 0.02 (SD = 0.65), comparison propensity scores following matching did not indicate a significant difference (p 0.89). Panels C and D are a comparison of propensity scores for matched IC group patients (y-axis) and control group (x-axis) utilizing a caliper of 1000 (matches every case to a control subject) and a caliper of 0.2 for PSM, respectively. With a caliper of 0.2, the paired differences between propensity scores for the IC and control groups were 0.01 (SD = 0.04), respectively.

Of the 814 patients enrolled or initially reviewed, 558 patients (280 IC tested and 278 untested control patients) met the inclusion and data requirements to be included in the analysis populations (Fig 1). A comparison of the clinical characteristics of the included and excluded IC tested patients are shown in S2 Table, where 8 of 9 factors are not different. The differing factor was nodule size with 12 (SD = 4) mm and 13 (SD = 5) mm (p 0.01) for included and excluded patients respectively. A comparison of patient and nodule characteristics of the IC tested and control patients before and after PSM is shown in Table 1; before and after PSM metrics are displayed in Fig 2. Prior to matching, the two cohorts differed in age, sex, smoking status, nodule size, nodule type, nodule location, and edge characteristics. Most notably, the prevalence of cancer was greater in the control population compared to the IC tested population (32% vs. 14%, p value <0.001) before matching. However, after 1:1 PSM, the distribution of clinical variables was similar across the 197 patients in each group (Table 1).

**Table 1:** Patient and nodule characteristics among IC tested and control groups before and after propensity match scoring.

CHARACTERISTICS	BEFORE PROPENSITY SCORE MATCHING			AFTER PROPENSITY SCORE MATCHING		
	IC Tested (n = 280)	Control (n = 278)	p value	IC Tested (n = 197)	Control (n = 197)	p value
Definitive Diagnosis, % (n)			<0.001			0.90
Benign	86% (n = 242)	68% (n = 188)		82% (n = 162)	82% (n = 161)	
Cancer	14% (n = 38)	32% (n = 90)		18% (n = 35)	18% (n = 36)	
Sex, % (n)			0.03			0.92
Male	38% (n = 107)	47% (n = 132)		45% (n = 89)	45% (n = 88)	
Female	62% (n = 173)	53% (n = 146)		55% (n = 108)	55% (n = 109)	
Age (years)			<0.01			0.91
Mean (SD)	68 (9)	65 (9)		67 (9)	66 (9)	
Range	42–90	37–87		42–90	41–87	
Smoking Status, % (n)			0.04			0.38
Current/Former	75% (n = 210)	82% (n = 228)		82% (n = 161)	78% (n = 154)	
Never Smoker	25% (n = 70)	18% (n = 50)		18% (n = 36)	22% (n = 43)	
Nodule Size (mm)			0.02			0.57
Mean (SD)	12.0 (3.9)	12.8 (4.4)		12.6 (4.3)	12.3 (4.2)	
Range	8–25	8–29		8–25	8–29	
Nodule Type, % (n)			<0.001			0.75
Solid	74% (n = 208)	60% (n = 167)		68% (n = 133)	66% (n = 130)	
Nodule Spiculation, % (n)			0.02			0.68
Spiculated	11% (n = 32)	19% (n = 52)		15% (n = 29)	16% (n = 32)	
Nodule Location, % (n)			0.21			0.54
Upper Lobe	45% (n = 126)	50% (n = 140)		44% (n = 87)	47% (n = 93)	
Prior Cancer, % (n)			0.27			0.76
Yes	5% (n = 13)	3% (n = 8)		3% (n = 5)	3% (n = 6)	

**Fig 3:**
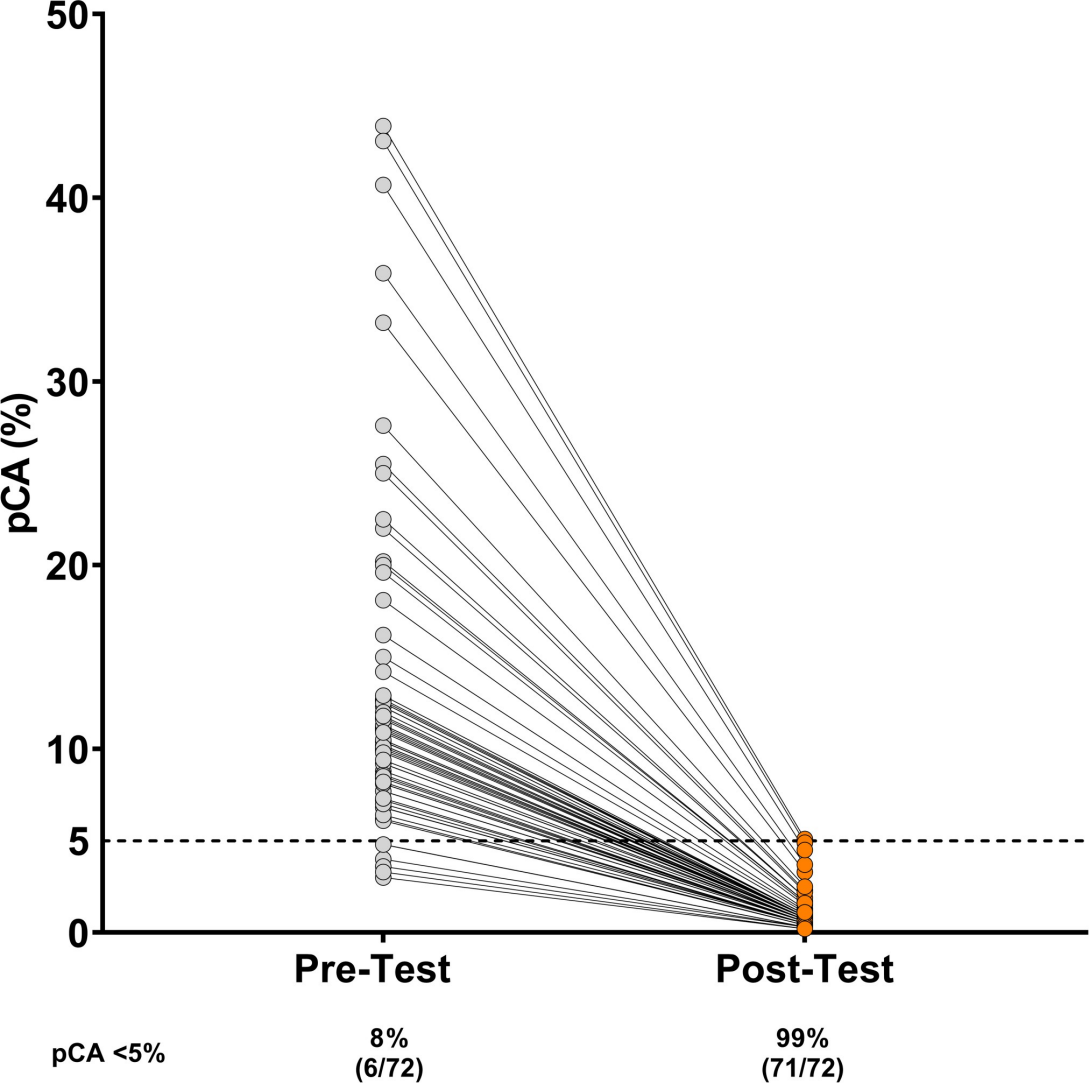
Comparison of Pre- and Post-Integrated Classifier (IC) risk distribution for Likely Benign test results. Distribution of the pre-test risk pCA and post-test risk classifications for IPNs classified as “Likely Benign” by the IC test in the PSM IC Tested group. Prior to IC Testing, 8% (6/72) of patients had a pre-test risk <5% pCA, following testing, 99% (71/72) of patients with a Likely Benign test result had a post-test risk <5% pCA. Indicating that 90% (65/72) of patients with Likely Benign test results were reclassified from the 5–65% risk category to the <5% risk category.

Of the 197 IC group patients evaluated with the IC test, 162 (82%) were benign and 35 (18%) were malignant. Following testing, 37% (72/197) of patients were classified as “Likely Benign” by the IC test, resulting in 92% (66/72) of patients with a Likely Benign result being reclassified to <5% pCA category from the 5–65% pCA risk category (Fig 3). Additionally, of the 72 patients with a Likely Benign test result, 89% (64/72) were directed to CT surveillance as the next action.

## Clinical outcomes

**Fig 4:**
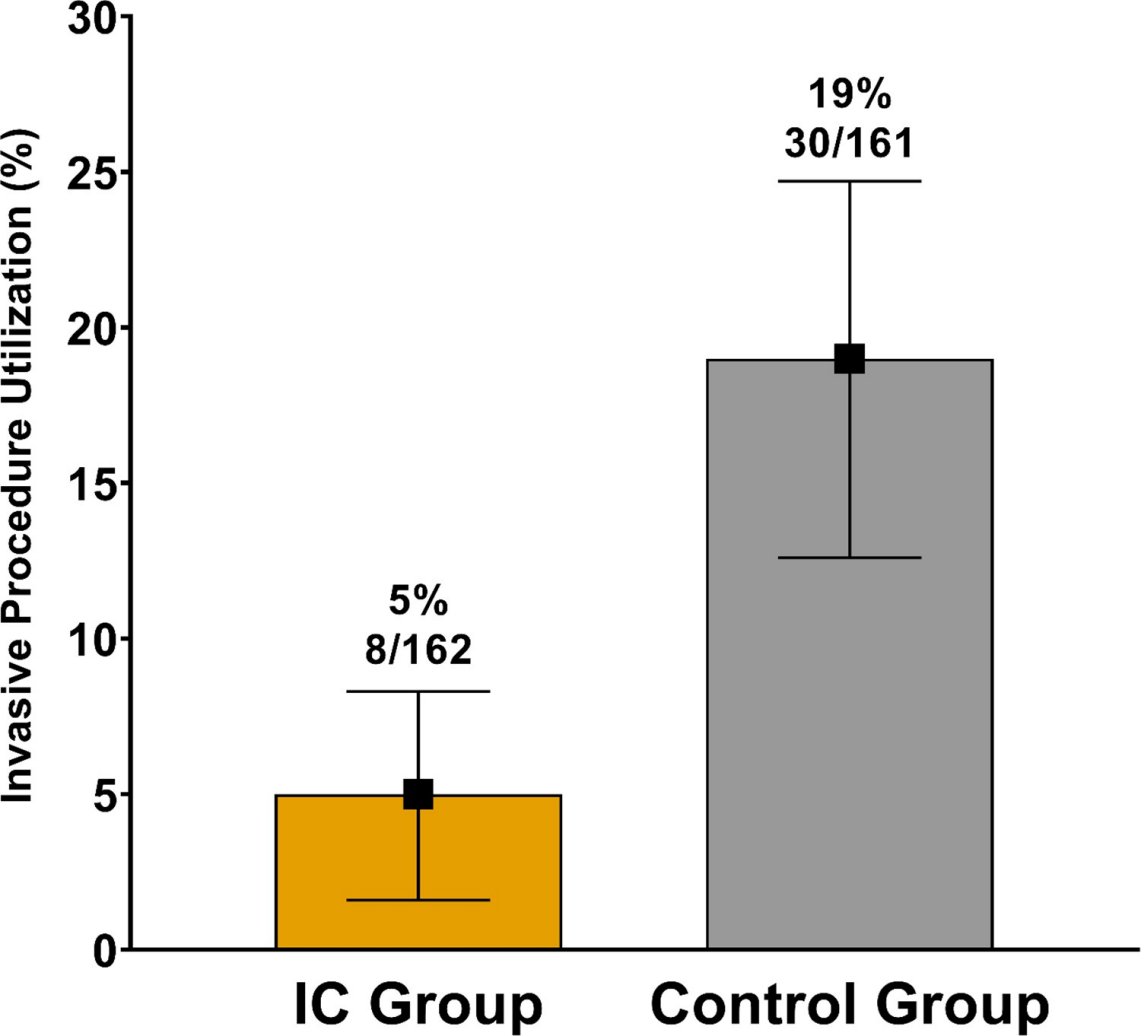
Invasive procedure utilization for patients with benign nodules managed with or without the Integrated Classifier (IC). The utilization of invasive procedures for patients with benign nodules was compared between the PSM IC and control groups. In the IC group, the proportion of invasive procedures for benign nodules was 5% (95% CI 1.6% to 8.3%, n = 8/162), whereas in the control group it was 19% (95% CI 12.6% to 24.7%, n = 30/161), with an absolute difference, 14%, (95% CI -19.5% to -7.9%) p <0.001, and relative reduction 74%.

Patients with a benign nodule in the IC group underwent fewer invasive procedures (n = 8, 5%) compared to patients in the untested control group (n = 30, 19%), yielding an absolute difference of -14%, [95% CI -19.5% to -7.9%], p <0.001 and relative reduction of 74%. (Fig 4 and Table 2), indicating a statistically significant reduction in invasive procedures on benign PNs. Moreover, of those patients with a benign diagnosis undergoing invasive procedures, 4 (2%) in the control group had surgical resection for their nodule, whereas no patients in the IC group managed with the IC test underwent surgical resection. Of the patients with a benign nodule in the IC group (n = 162), there was one invasive procedure per twenty patients as opposed to one invasive procedure per five patients in the control group (n = 161); odds ratio of 0.23, [95% CI 0.09 to 0.53], p <0.001. Thus, for every 7 benign nodules tested with the IC, one unnecessary invasive procedure was avoided.

**Table 2:** Proportion of invasive procedures performed on benign lung nodules between the PSM IC and control groups.

Category	IC group (Benign) (n = 162)	Control group (Benign) (n = 161)	Absolute Difference	P value	Relative Difference
**`Invasive Procedures**	5% (n = 8)	19% (n = 30)	-14%	<0.001	-74%
**(95% CI)**	(1.6% to 8.3%)	(12.6% to 24.7%)	(-19.5% to -7.9%)		
**Biopsies**	5% (n = 8)	17% (n = 27*)	-12%	<0.001	-71%
**(95% CI)**	(1.7% to 8.6%)	(11.0% to 22.6%)	(-13.3 to -6.0%)		
**Surgeries**	0 (0%)	2% (n = 4)	-2%	0.002	NA
**(95% CI)**		(0.8% to 4.2%)	(-3.9% to -1.0%)		

*one patient with a benign nodule underwent both a biopsy and surgical resection

The proportions of PNs followed by CT surveillance resulting in a malignant diagnosis in the IC Tested and control groups (a potential false negative and safety concern) were evaluated in the larger analysis populations and the PSM groups **(**Fig 1**)**. Prior to PSM, 236 patients in the IC tested analysis population were initially routed to CT surveillance, with 14 (5.93%) patients eventually diagnosed with a malignancy. Similarly, in the control analysis population, 139 patients were sent to CT surveillance by usual care, with 10 (7.19%) patients eventually being diagnosed with a malignancy. When these two rates were compared, the absolute difference was -1.26% [95% CI -5.67% to 3.14%], p 0.319, indicating no statistically significant difference between the two analysis populations. Similarly, when the PSM groups were evaluated, 161 patients routed to CT surveillance in the IC tested group, with 12 (7.45%) patients eventually diagnosed with a malignancy. The control group, with 113 patients routed to CT surveillance, had 4 (3.53%) patients eventually diagnosed with a malignancy. When these two rates were compared, the absolute difference was 3.91% [95% CI -0.53% to 8.36%], p 0.075, indicating that the difference was not statistically significant. Overall, in both the analysis populations and the PSM groups, the rates of surveilled malignant nodules did not statistically differ between the IC tested and control groups.

## Discussion

This study, using the proteomic IC, adds to our knowledge of how physicians manage pulmonary nodules in several important ways. First, 37% of patients in the IC group had an actionable likely benign result, classifying the probability of cancer from ≤ 50% to < 5%. Second, physician management of intermediate risk nodules changed such that in patients with benign nodules, invasive procedures for patients were significantly reduced. Finally, although a small percentage of nodules routed to surveillance eventually were malignant, the rate when using the biomarker was not significantly different when compared to management with usual care.

Lung nodules often pose a diagnostic challenge for clinicians. When presented with a newly observed nodule, the goal of the clinician is to avoid invasive procedures in those nodules that are ultimately found to be benign, and in those nodules that are malignant, move patients towards treatment with curative intent without delay. The decision on management is usually based on physician or model derived pre-test pCA, the local availability and expertise of the imaging test or procedure, and patient preference [2, 16]. In most cases it is recommended that those with a low pCA (< 5%) be followed with CT surveillance for 2 years to confirm benignity and those with a high pCA (> 65%) undergo biopsy and surgical resection. In the group in between (pCA 5% - 65%) further testing with either PET scan, bronchoscopy or TTNB is recommended. By current estimates ~84% of PN’s fall into this intermediate range where the tools and invasive procedures to aid in distinguishing benign from malignant disease have remained largely unchanged for at least the last decade [17].

How significant is the issue of invasive procedures in those with benign lung nodules? Tanner et al, studied 377 patients in 18 geographically diverse community pulmonary practices where the prevalence of malignancy was 25% [17]. In that study, 41% of patients with benign nodules (116/283) underwent an invasive procedure. Of greater concern, 35% percent of patients referred for surgical resection had benign disease and the rate of surgical resection was similar among those with low, intermediate, or high-risk nodules, suggesting clinicians did not always follow guideline directed care. These observations have been reinforced in a report from a large academic center, which showed that 1 in 5 incidentally identified PNs did not receive guideline concordant care [18]. The recent CHEST guidelines on lung cancer screening confirmed these findings in a compilation of prospective lung cancer screening studies [19]. Combining data on lung nodules discovered on screening CT, Mazzone and colleagues reported that 37% of patients underwent invasive procedures (bronchoscopy or TTNB), and 22% had surgical resection for benign lung nodules. Kammer and colleagues also recently reported a 63% rate of invasive procedures in those with benign nodules [20]. However, there are recent reports indicating improvement in guideline-concordant care [21]. Nonetheless, invasive procedures and thoracic surgery are not without complications and these data support the need for additional tools to better manage patients with undiagnosed PN’s.

The IC studied here has transitioned from discovery to validation studies and now we report clinical utility [8–10]. The development of this plasma-based proteomic IC followed a pathway outlined by Mazzone et al., for evaluating molecular biomarkers and assessing when one should be considered ready for clinical use [22, 23]. After completion of the discovery and validation studies as recommended, a prospective, multicenter observational trial of patients with 8–30 mm PN’s was undertaken to validate the test characteristics of the biomarker [9]. A subgroup of 178 patients was identified with the greatest opportunity for reducing invasive procedures on benign nodules. This population had a lung cancer prevalence of 16% and a pretest pCA ≤ 50% [9]. The IC demonstrated a sensitivity of 97%, a specificity of 44%, and a negative predictive value of 98% in distinguishing benign from malignant nodules. The IC performed better than PET, validated lung nodule risk models, and physician cancer probability estimates at differentiating benign from malignant disease. If the IC results were used to direct care, 40% fewer procedures could have been performed on benign nodules, while 3% of malignant nodules would be misclassified as benign [9].

This PSM clinical utility study, as opposed to a randomized controlled trial, does not have a prospective control group, so comparisons must be made using historical cohorts, by retrospective review of standard of care, or by comparing intended procedures to actual procedures. In a recent publication describing a biomarker used after non-diagnostic bronchoscopy, physician procedure plans before a test result were compared to the actual procedures informed by test results [24]. Here, use of invasive procedures was evaluated before and after the introduction of the test at each study site using PSM methodology to ensure that valid comparisons could be made between groups. In this report, of 197 IC Tested patients managed by the integrated classifier, nearly 40% had an actionable result, and there was a relative reduction of invasive procedures by 74%. Whereas in the untested Control Group, 19% of benign patients underwent an invasive procedure, with 4 (2%) patients routed to surgery.

It is remarkable that the invasive procedures on patients with benign nodules in this study are lower compared to prior reports. For example, the rates of procedures in the Tanner study were 41% and the current rates are only 19% and 5% for the control and IC tested groups respectively [18]. Similarly, the rates of malignant nodules routed to CT surveillance are also lower in this study. For comparison, two studies have reported that 13% and 11% of PNs sent to CT surveillance result in a malignant diagnosis [9, 17, 25]. In this study, prior to PSM, the proportions of surveilled malignant nodules are 6% and 7% in the IC tested and control analysis population, respectively. After PSM, these proportions remain small at 7% and 4%, in the IC tested and control groups, respectively. Although, the PSM process does impact the proportions of surveilled malignant PNs as compared to the overall analysis population, neither group showed a significant difference. The reasons for improvement compared to historical data are not known but could be due to more guideline-concordant care, selection of sites with structured nodule evaluation programs, more experience, or participation in a clinical trial. Additionally, these proportions could also be impacted by unmeasured factors that affect classification of nodules not measured in this study, such as body mass index [26].

The use of the IC test reduced the likelihood that a patient would undergo an unnecessary invasive procedure on a benign PN. This documented reduced likelihood needs to be balanced against the potential risk of a delayed diagnosis of cancer. The risk of a delay in diagnosis was evaluated in a recent systematic review and concluded that precise quantification was not currently possible [27]. Since that review, a study with a large veterans’ population found adverse outcomes with Stage I lung cancer if surgery was delayed more than 12 weeks [28]. Also, a large review of seven cancer types and delays on mortality, used prioritization and modeling to conclude that even a 4-week delay was concerning [29]. A delayed cancer diagnosis in a PN should continue to be mitigated by the backstop of CT surveillance, as recommended by the ACCP guidelines [2]. It is recommended that CT scan follow-up intervals remain based on the pre-test probability rather than the post-test probability so that patients whose nodules are reclassified to low risk will still have the benefit of timely monitoring.

This study has several limitations. First, definitive diagnoses for nodules that were surveilled in this analysis were based on one year of follow-up. Thus, a nodule could be misclassified here as benign when it might have subsequently been malignant. This seems unlikely as shown in the previous validation trial, which initially reported data at one year, and recently showed that there was no change in diagnoses at 2 years, making it unlikely that these results will significantly change over time [9, 30]. This is an observational rather than interventional study so there is variation in management and follow-up, though the findings from the ORACLE study will be more representative of real-world practice than past observational studies without the use of the biomarker. An additional limitation of this study was the lack of randomization to evaluate the impact of the IC test on clinical practice. Using a historical control cannot eliminate bias such as secular trends or other changes in physician’s behavior. This study addressed this potential by selecting patients at the same practices that were treated prior to the initiation of this study and by using a standard propensity score matching process to balance clinical characteristics between the groups. Finally, the consequence for misclassifying patients with malignancy to likely benign and placing them in surveillance is unknown. Importantly, the rate that this occurred was not statistically different than the control group. However, this study has a relatively small sample of surveilled malignant nodules, and the results could be different with a larger study. To further evaluate the clinical utility of the IC test, a large randomized controlled trial (the ALTITUDE trial—NCT04171492) using this IC versus usual care for the management of nodules is underway and should provide additional detail for these limitations. Also, the misclassification with IC testing is not worse than other tools used for nodule classification, such as PET [31]. Furthermore, a study by Maiga et al., evaluating the performance of PET scans for risk stratifying high-risk lung nodules identified that the negative predictive value of PET scans can vary significantly, ranging from 17–70% [31]. This indicates that a negative PET scan does not always correspond to a benign designation. We recommend the approach of using the IC to supplement clinical factors and to assist in clinical decision making, rather than it being seen as the sole determinate for diagnosis or patient management. Still, it is essential to note that a likely benign IC test result still requires follow-up with surveillance CT, and should growth be detected on a subsequent scan, appropriate diagnostic action is indicated.

This study has several strengths including the real-world study population, an analysis utilizing PSM as opposed to unmatched historical controls, rigorous laboratory standards, and completion of enrollment before the coronavirus 2019 (COVID-19) pandemic. Furthermore, the PSM approach utilized was able to maximize the number of IC tested patients matched to control subjects (~70%) and reduced the imbalance among the covariates most likely to be associated with invasive procedure utilization. The study population is from community clinics (73%) and academic centers (27%) and includes only patients with ≤ 3 cm nodules, whereas other biomarker studies have included over 20% of lesions > 3 cm, which no longer fit the diagnostic criteria of nodules [24]. Moreover, sampling and testing in this study were all done in real-time, rather than on stored or archived samples. The test algorithm has been locked since 2016, and test result reporting uses numerical values for more precise information compared to descriptive or categorical results. Finally, study enrollment was completed at the onset of the COVID-19 pandemic, avoiding effects on patient referrals and enrollment.

### Conclusions

This proteomic blood-based IC for newly discovered lung nodules has demonstrated clinical utility in a pragmatic, real-world clinical setting. Use of the test shows a relative reduction of invasive procedures on benign nodules by 74%. Reductions in invasive procedures on benign nodules may lead to improvement in patient outcomes, by reducing adverse events, complications, and hospitalizations associated with invasive procedures, decreasing anxiety for the patient, and decreasing costs to both the patient and the health care system.

## Supporting information

S1 Checklist STROBE statement—Checklist of items that should be included in reports of *cohort studies*. (DOCX)

S1 Table ORACLE site list and investigators. (DOCX)

S2 Table Comparison of clinical characteristics between included cases and excluded patients. (DOCX)

S1 Data (XLSX)

S1 File (PDF)
