# Supplementary material for: Assessing a biomarker’s ability to reduce invasive procedures in patients with benign lung nodules: Results from the ORACLE study
Source: PLoS One. 2023 Jul 11;18(7):e0287409. doi: 10.1371/journal.pone.0287409 (PMC10335667; doi:10.1371/journal.pone.0287409)
Supplement: S1 Table — (DOCX) [file pone.0287409.s002.docx]

**S1 Table. ORACLE site list and investigators***

| **Site Name** | **City** | **State** | **Site Primary Investigator** |
| --- | --- | --- | --- |
| Pinehurst Medical Clinic | Pinehurst | NC | Michael Pritchett, DO, MPH |
| Stamford Health | Stamford | CT | Michael Bernstein, MD |
| Southeastern Research Center | Winston-Salem | NC | Barry Sigal, MD |
| Oregon Clinic | Portland | OR | Melvin Morganroth, MD |
| University of Nevada, Las Vegas | Las Vegas | NV | Arthur Romero, MD |
| Eastern Carolina University | Greenville | NC | Mark Bowling, MD |
| University of Cincinnati | Cincinnati | OH | Alejandro Aragaki, MD |
| Sarasota Memorial | Sarasota | FL | Joseph Seaman, MD |
| Banner Health/MD Anderson Cancer Center | Sun City | AZ | Archan Shah, MD, MRCP |
| Medical College of Wisconsin | Milwaukee | WI | Jonathan Kurman, MD |
| PeaceHealth | Bellingham | WA | Donald Berry, MD |
| Pueblo Pulmonary Associates | Pueblo | CO | Josiah Gordon, DO |
| Parkview Research Center | Fort Wayne | IN | Abhishek Biswas, MD |
| Clinical Research Associates of Central PA/Penn Highlands Hospital | DuBois | PA | Sandeep Bansal, MD |

*Sites listed both enrolled patients and submitted clinical data for the study analyses
