# Supplementary material for: Assessing a biomarker’s ability to reduce invasive procedures in patients with benign lung nodules: Results from the ORACLE study
Source: PLoS One. 2023 Jul 11;18(7):e0287409. doi: 10.1371/journal.pone.0287409 (PMC10335667; doi:10.1371/journal.pone.0287409)
Supplement: S2 Table — (DOCX) [file pone.0287409.s003.docx]

**S2 Table: Comparison of Clinical Characteristics Between Included Cases and Excluded Patients***

|  | patients included in IC Tested Analysis Population  (n=280) | Patients excluded from IC Tested Analysis Population (n=139) | p value |
| --- | --- | --- | --- |
| Sex, % (n) |  |  | 0.49 |
| Male | 38% (n=107) | 42% (n=58) |  |
| Female | 62% (n=173) | 58% (n=81) |  |
| Age (years) |  |  | 0.67 |
| Mean (SD) | 68 (9) | 67 (9) |  |
| Range | 42-90 | 42-92 |  |
| Smoking Status, % (n) |  |  | 0.90 |
| Current/Former | 75% (n=210) | 76% (n=105) |  |
| Never Smoker | 25% (n=70) | 24% (n=34) |  |
| Nodule Size (mm) |  |  | 0.01 |
| Mean (SD) | 12 (4) | 13 (5) |  |
| Range | 8-25 | 8-29 |  |
| Nodule Type, % (n) |  |  | 0.97 |
| Solid | 74% (n=208) | 74% (n=104) |  |
| Nodule Spiculation, % (n) |  |  | 0.51 |
| Spiculated | 11% (n=32) | 14% (n=19) |  |
| Nodule Location, % (n) |  |  | 0.95 |
| Upper Lobe | 45% (n=126) | 45% (n=63) |  |
| Prior Cancer, % (n) |  |  | 0.43 |
| Yes | 5% (n=13) | 6% (n=9) |  |

*Analysis performed to evaluate if patients excluded from analyses differ from the included cases
