# Supplementary material for: Assessing a biomarker’s ability to reduce invasive procedures in patients with benign lung nodules: Results from the ORACLE study
Source: PLoS One. 2023 Jul 11;18(7):e0287409. doi: 10.1371/journal.pone.0287409 (PMC10335667; doi:10.1371/journal.pone.0287409)
Supplement: S1 File — (PDF) [file pone.0287409.s005.pdf]

Clinical Protocol Number BDX-CD-002

An Observational Registry Study to Evaluate the Performance of the BDX-XL2 Test

(ORACLE)

Version 1.1
06 AUG 2018

Biodesix, Inc.
219 Terry Avenue North, Suite 100
Seattle, WA 98109

Sponsor Principal Investigator
Linda Traylor, PhD

CONFIDENTIAL AND PROPRIETARY INFORMATION

The information contained within this document may not be used, divulged, published, or otherwise disclosed without prior written consent of Biodesix.

SPONSOR SIGNATURE PAGE

Protocol Name:	ORACLE Registry
Protocol Number:	BDX-CD-002
Date:	06 AUG 2018
Version:	1.1
Title:	An Observational Registry Study to Evaluate the Performance of the BDX-XL2 Test
Test Product:	BDX-XL2
Clinical Laboratory:	Biodesix, Inc.
Sponsor:	Biodesix, Inc.<br>219 Terry Ave. North, Suite 100<br>Seattle, WA 98109

The signatures of the Sponsor below constitute approval of this protocol and provide the necessary assurances that this study will be conducted according to all conditions of the protocol, the Investigator Contractual Agreements and applicable laws and regulations, including all statements regarding confidentiality.

Linda Traylor, PhD
Vice President, Clinical Development and Medical Affairs

Signature:____ Date:_______________

CONFIDENTIAL AND PROPRIETARY INFORMATION

The information contained within this document may not be used, divulged, published, or otherwise disclosed without prior written consent of Biodesix.

SITE INVESTIGATOR SIGNATURE PAGE

Protocol Name:	Oracle Registry
Protocol Number:	BDX-CD-002
Date:	06 AUG2018
Version:	1.1
Title:	An Observational Registry Study to Evaluate the Performance of the BDX-XL2 Test
Test Product:	BDX-XL2
Clinical Laboratory:	Biodesix, Inc.
Sponsor:	Biodesix<br>219 Terry Ave. North, Suite 100<br>Seattle, WA 98109

The signature of the Principal Investigator below constitutes approval of this protocol and provides the necessary assurances that this study will be conducted according to all conditions of the protocol, the Investigator Contractual Agreements and applicable laws and regulations, including all statements regarding confidentiality.

Site Number:________________

Site Name:____

Principal Investigator:

Printed Name:____

Signature:____ Date:________________

CONFIDENTIAL AND PROPRIETARY INFORMATION

The information contained within this document may not be used, divulged, published, or otherwise disclosed without prior written consent of Biodesix.

SPONSOR CONTACT INFORMATION

Biodesix may be contacted at the following:

Linda Traylor, PhD
Vice President, Clinical Development & Medical Affairs
(303) 656-5367
linda.traylor@biodesix.com

Niki Givens
Sr. Director, Clinical Research
(720) 495-1583
niki.givens@biodesix.com

Russell Hudnall
Director of Clinical Operations
(206) 576-6388
russell.hudnall@biodesix.com

CONFIDENTIAL AND PROPRIETARY INFORMATION

The information contained within this document may not be used, divulged, published, or otherwise disclosed without prior written consent of Biodesix.

Table of Contents

1

1. Background and Rationale 12
1.1 Introduction 12
1.2 Relevant Literature and Data 12
1.2.1 Survey and Chart Review 12
1.2.2 Development and Validation of BDX-XL2 13
1.3 Comparative Performance of BDX-XL2 to Current Practices 13
1.3.1 Current Risk Predictor Selections for Comparison to BDX-XL2 13
1.4 Technical Definition of BDX-XL2 14
1.5 Reporting BDX-XL2 Results 14
1.6 Intended Use Population 14

2. Study Objectives 14
2.1 Primary Objective 15
2.2 Secondary Objectives 15
2.3 Exploratory Objectives 15

3. Statistical Analysis 15
3.1 Study Population 15
3.2 Sample Size Considerations 15

4. Study Plan 16
4.1 Registry Design 16
4.1.1 Description of Participating Sites 16
4.1.2 Overview of Data Collection 16
4.1.3 Overview of Biospecimen Collection 17
4.1.4 Study Duration, Enrollment and Number of Sites 17
4.1.5 Inclusion Criteria 18
4.1.6 Exclusion Criteria 18
4.2 Registry Procedures 18
4.2.1 Registry Timepoints 18

CONFIDENTIAL AND PROPRIETARY INFORMATION

The information contained within this document may not be used, divulged, published, or otherwise disclosed without prior written consent of Biodesix.

4.2.2 Patient Enrollment 18
4.2.3 Post-Test Result Visit 19
4.2.4 Follow-Up Timepoints 20
4.2.5 Discontinuation 21
4.3 Medical Data Collection Procedures 22
4.3.1 Data Collection 22
4.3.2 Protected Health Information Collected 22

5. Study Administration 23
5.1 Study Oversight 23
5.1.1 Site Qualification Visits 23
5.1.2 Site Initiation Visits 23
5.1.3 Routine Monitoring Visits 23
5.1.4 Close Out Visits 24
5.2 Regulatory Oversight 24

6. Data Handling and Quality Assurance 25
6.1 Electronic Systems 25
6.2 Confidentiality of Subjects 25
6.3 Biospecimen Collection and Management 25
6.4 Reporting Results to Subjects 25

7. Regulatory and Ethical Considerations 25
7.1 Risk Assessment 25
7.2 Potential Benefits of Participation 25
7.3 Recruitment Strategy 26
7.4 Informed Consent 26
7.5 Payments to Patients 26
7.6 Confidentiality 26

8. Safety Management 26
8.1 Clinical Adverse Events 26
8.2 Safety Events 27

CONFIDENTIAL AND PROPRIETARY INFORMATION

The information contained within this document may not be used, divulged, published, or otherwise disclosed without prior written consent of Biodesix.

8.3 Safety Event Reporting 27

9. Publication Plan 27

10. References 27

CONFIDENTIAL AND PROPRIETARY INFORMATION

The information contained within this document may not be used, divulged, published, or otherwise disclosed without prior written consent of Biodesix.

Synopsis:

Title	An Observational Registry Study to Evaluate the Performance of the BDX-XL2 Test
Study Design	A multicenter, observational research registry study to evaluate the Impact of the BDX-XL2 integrated classifier when used in the clinical management of recently identified lung nodules assessed to have a low to moderate risk of cancer.
Study Sponsor	Biodesix
Study Sites	Approximately 10
Subject Population	N=500
Therapeutic Intervention	None
Device Use	None
Procedures	Venous Blood Collection
Subject Safety Issues	• Minimal Risk – Only clinical risk related to the study are those associated with routine venous phlebotomy.<br>• Monitoring for Safety Events (as defined by the protocol) will be conducted throughout the duration of the registry.
Primary Study Objective	To show a statistically significant reduction in the proportion of benign lung nodules experiencing invasive procedures (biopsies or surgery) between a prospective group of patients managed by BDX-XL2 and a contemporaneous group not managed by BDX-XL2.
Secondary Study Objectives	To show BDX-XL2statistically significant noninferiority of the proportion of malignant lung nodules routed to CT surveillance, preventing a potentially curative resection, under management of lung nodules by BDX-XL2 as compared to contemporaneous group not managed by BDX-XL2.<br>To show statistically significant noninferiority of BDX-XL2 potentially curative resections of malignant nodules under management of lung nodules by BDX-XL2 as compared to a contemporaneous group not managed by BDX-XL2.
Exploratory Objectives	To evaluate and compare the standard of care risk assessment models.<br>To evaluate and compare the patterns of healthcare utilization between a group managed with BDX-XL2 and a contemporaneous group not managed by BDX-XL2.

CONFIDENTIAL AND PROPRIETARY INFORMATION

The information contained within this document may not be used, divulged, published, or otherwise disclosed without prior written consent of Biodesix.

Inclusion Criteria	1. Patient has provided informed consent to participate in the registry and agrees to comply with all protocol requirements.<br>2. Patient meets the criteria for the intended use population of the BDX-XL2 test:<br>• Patient is ≥ 40 years of age at the time of the discovery of the lung nodule of concern.<br>• The maximal dimension of the patient's lung nodule of concern is ≥ 8mm and ≤ 30mm.<br>• The pre-test risk of cancer as determined by the Mayo risk prediction algorithm is 50% or less.<br>3. The first CT scan identifying the lung nodule of concern was performed within 60 days of patient enrollment in the registry.
Exclusion Criteria	1. Nodule work-up before the time of patient enrollment indicating any prior attempted or completed diagnostic procedure.<br>2. Current diagnosis of any active cancer.<br>3. High risk per physician assessment.<br>4. Prior diagnosis of any cancer within 2 years of lung nodule detection, except for non-melanoma skin cancer.<br>5. Concurrent participation in any unrelated clinical trial that may impact or alter the management of the patient's nodule of concern.<br>6. Any illness or factor that will prevent compliance with follow-up as recommended.
Statistical Methods	There will be two defined study populations<br>i. Overall study population including all patients enrolled in the ORACLE registry<br>ii. Primary and Second Endpoint analysis population including all subjects enrolled in the ORACLE registry with definitive diagnoses of their identified nodules either by histopathology or radiology (stability or nodule resolved).<br>Formal statistical comparison of primary and secondary endpoints will be carried out with the Primary and Secondary Endpoint analysis population using appropriate statistical tests and methods prespecified in the formal statistical analysis plan (SAP). Statistical methods used for exploratory endpoints will be primarily descriptive in nature. Categorical variables will be summarized using frequency counts and percentages. Continuous variables will be summarized

CONFIDENTIAL AND PROPRIETARY INFORMATION

The information contained within this document may not be used, divulged, published, or otherwise disclosed without prior written consent of Biodesix.

	by total number of observations, mean, standard deviation, median and range.<br>A formal SAP will be developed and finalized prior to statistical analysis of any study data. It will fully specify all statistical analyses to be performed along with the statistical methods to be used.

CONFIDENTIAL AND PROPRIETARY INFORMATION

The information contained within this document may not be used, divulged, published, or otherwise disclosed without prior written consent of Biodesix.

Abbreviations

AE	Adverse Event
CMS	Centers for Medicare & Medicaid Services
COV	Close Out Visit
CRF	Case Report Form
CT	Computed Tomography
EDC	Electronic Data Capture
EMR	Electronic Medical Records
GCP	Good Clinical Practices
GML	Guardian Medical Logistics
ICF	Informed Consent Form
ISF	Investigator Site File
LCD	Local Coverage Determination
LIS	Laboratory Information System
NPV	Negative Predictive Value
pCA	Predicted Risk of Cancer
PET	Positron Emission Tomography
PHI	Protected Health Information
PI	Principal Investigator
RMV	Routine Monitoring Visit
SAE	Serious Adverse Event
SIV	Site Initiation Visit
SMF	Site Master File
SOC	Standard of Care
SQV	Site Qualification Visit
TMF	Trial Master File
TRF	Test Request Form
TTNA	Transthoracic Needle Aspiration

CONFIDENTIAL AND PROPRIETARY INFORMATION

The information contained within this document may not be used, divulged, published, or otherwise disclosed without prior written consent of Biodesix.

1. Background and Rationale

1.1 Introduction

It is estimated that the number of new lung nodules incidentally detected in the U.S. is 1.57 million annually [1]. In addition, the detection of another 1.5 million new lung nodules annually in the US is projected with the recent Medicare coverage for lung cancer screening. The evaluation and management of 3 million new lung nodules annually will require more robust resources and will benefit from additional guidance and tools.

Evaluation of lung nodules poses physicians with the challenge of estimating the probability of cancer and choosing the appropriate management path. Depending on probability of cancer, patients with very low risk nodules may be managed with computerized tomography (CT) surveillance, whereas patients with high-risk nodules may proceed directly to definitive therapy with surgical excision. Management for the remaining group of patients with nodules in the "intermediate risk" for cancer spectrum frequently involves additional diagnostic testing [including positron emission tomography (PET), CT- guided transthoracic needle biopsy (TTNA) and/or bronchoscopy] [1]. However, as the majority of identified lung nodules are benign, the use of unnecessary invasive and diagnostic procedures during patient work-up is quite prevalent [2].

Prior to its acquisition by Biodesix, Inc., Integrated Diagnostics, Inc. (Indi) developed a lung nodule classifier now referred to as BDX-XL2, a liquid biopsy that offers physicians an integrated classifier as an additional tool in the management of lung nodules. When used in the evaluation of patients whose lung nodules are a low to moderate risk for cancer, BDX-XL2 accurately identified benign lung nodules in the PANOPTIC study with a prospective-retrospective study design. This blood test should provide physicians with additional assurance to correctly follow a CT surveillance path of management, while decreasing the risks and costs of avoidable diagnostic and invasive procedures. Based on PANOPTIC study results, Medicare CMS concluded that the BDX-XL2 assay is reasonable and necessary in the management of lung nodules by identifying those lung nodules with a high probability of being benign.

BDX-XL2 received a Local Coverage Determination (LCD) L37054 for Medicare beneficiaries. As noted in the LCD, participation in the ORACLE Registry is a mandated requirement for coverage of the BDX-XL2 assay for Medicare beneficiaries. As the sponsor of the ORACLE Registry, Biodesix has committed to working with participating physicians to ensure compliance with the registry requirements and to assist in gathering and reporting relevant information to Medicare.

1.2 Relevant Literature and Data

During the development of BDX-XL2, the developer worked with leading physicians and scientists to produce peer-review publications and presentations in major journals and meetings. This research has been conceived, designed, and conducted with collaboration of major leaders in the fields of molecular diagnosis, lung cancer, and pulmonary nodules. These efforts will be summarized here with details of results in the appended publications.

1.2.1 Survey and Chart Review

Indi contracted with Boston Healthcare for a survey of pulmonary physicians to determine their practice patterns and potential acceptance of a biomarker for pulmonary nodules. The need and acceptance was confirmed and published in 2014 [2].

Boston Healthcare also collected data for a comprehensive chart review of community pulmonary practices to understand practice patterns where most of pulmonary nodules are managed. Nodule management of 377 patients from 18 geographically diverse sites was assessed. The results have been

CONFIDENTIAL AND PROPRIETARY INFORMATION

The information contained within this document may not be used, divulged, published, or otherwise disclosed without prior written consent of Biodesix.

presented at national meetings and published in 2015 [7]. Of particular note was the finding that benign nodules had a 61% biopsy and 35% surgery rate establishing a clear unmet need.

1.2.2 Development and Validation of BDX-XL2

BDX-XL2 was developed on Study 1013 (NCT01752101) [3] and then verified and validated on the PANOPTIC study (NCT01752114) [4]. Study 1013 and PANOPTIC are both prospective studies of lung nodules, designed and sponsored by Integrated Diagnostics (now owned by Biodesix, Inc.). These studies are summarized in Table 1.

Table 1. Study 1013 and PANOPTIC showing development phases and numbers of sites.

	**Study 1013**	**PANOPTIC**
**Development Phase**	Discovery	Verification & Validation
**Number of Sites**	12	33
**Patients Enrolled**	475	685
**Intended Use Patients**	222	178

Development and validation of BDX-XL2 adhered to the best practices for test development as defined by the National Academy of Medicine (NAM) Guidelines for best practices in test development and validation [5]. In particular, discovery and validation were both prospective and conducted on a large number of independent sites. Additionally, verification and validation were conducted under a strict blinding protocol and utilized a 3rd party statistician. This is the highest level of clinical validation achievable by the NAM.

1.3 Comparative Performance of BDX-XL2 to Current Practices

1.3.1 Current Risk Predictor Selections for Comparison to BDX-XL2

Silvestri et. al [4] compares the performance BDX-XL2 to six other cancer risk predictors for lung nodules. All comparisons are made using the same 178 PANOPTIC patients in the lower risk, intended use population, allowing for a direct comparison. These six predictors fall into three categories as follows.

<u>Current Practice</u>: Current practice for estimating the cancer risk of a lung nodule is the initial physician cancer risk assessment (pCA) based on physician clinical judgement [6]. This also represents how cancer risk was estimated for over 80% of lung nodules evaluated in PANOPTIC and is also a practice recommended in the ACCP Guidelines.

<u>PET</u>: PET is referenced in the American College of Chest Physicians (ACCP) Guidelines for use as a tool for assessing cancer risk. PET was used in 75 of 178 (42%) of intended use subjects in the PANOPTIC study.

<u>Clinical Risk Predictors</u>: Four clinical risk predictors were assessed; Mayo [7], VA [8], Brock [9] and Herder [10]. Mayo and VA are referenced in the ACCP Guidelines; however, they were used by less than 20% of physicians in the PANOPTIC study. The Brock and Herder models are included for completeness but were not utilized by physicians in the PANOPTIC study and are not presented below.

CONFIDENTIAL AND PROPRIETARY INFORMATION

The information contained within this document may not be used, divulged, published, or otherwise disclosed without prior written consent of Biodesix.

1.4 Technical Definition of BDX-XL2

BDX-XL2 integrates the relative abundance of two plasma proteins (LG3BP and C163A) with five clinical risk factors (age, smoking status, nodule diameter, nodule spiculation status and nodule location). XL2 provides a numerical value, *XL_2*(k), for a subject k, as defined below:

$$XL\_2(k) = \begin{cases} \max(0, p(k) - 0.5), & log_2(\frac{LG3BP}{C163A}) \leq .38 \\ p(k), & log_2(\frac{LG3BP}{C163A}) > .38 \end{cases}$$

$$p(k) = \frac{e^X}{1 + e^X}$$

$$X = -6.8272 + 0.0391 * Age + 0.7917 * Smoker + 0.1274 * Diameter + 1.0407 * Spiculation + 0.7838 * Location$$

where *Age* is the age of the subject in years, *Smoker* is 1 if the subject is a former or current smoker (otherwise 0), *Diameter* is the size of the lung nodule in mm, *Spiculation* is 1 if the lung nodule is spiculated (otherwise 0) and *Location* is 1 if the lung nodule is located in an upper lung lobe (otherwise 0). The linear function X that integrates the clinical risk factors is a simplification of the Mayo clinical risk predictor [7] that eliminates the cancer risk history factor.

XL_2(k) ranges between 0 and 1. The closer *XL_2*(k) is to 0, the more likely subject k has a very high NPV which is calculated using PANOPTIC data. (See Appendix 2 for results of the PANOPTIC study)

1.5 Reporting BDX-XL2 Results

BDX-XL2 results will be reported as Likely Benign or Indeterminate.

1.6 Intended Use Population

The intended use population of BDX-XL2 are patients:

- $\geq$ 40 years of age
- with a lung nodule with diameter of 8mm to 30mm
- with a pre-test risk of cancer (as determined by the Mayo risk prediction algorithm) of 50% or less.

2. Study Objectives

The ORACLE Registry study is being conducted to demonstrate clinical utility of the BDX-XL2 test; specifically, the potential to reduce unnecessary invasive procedures, such as biopsies and surgeries, on benign lung nodules while not significantly increasing the number of malignant lung nodules routed to CT surveillance.

CONFIDENTIAL AND PROPRIETARY INFORMATION

The information contained within this document may not be used, divulged, published, or otherwise disclosed without prior written consent of Biodesix.

2.1 Primary Objective

1. The primary objective of this study is to show a statistically significant reduction in the proportion of benign lung nodules experiencing invasive procedures (biopsies or surgery) between a prospective group of patients managed by BDX-XL2 and a contemporaneous group not managed by BDX-XL2.

2.2 Secondary Objectives

1. The secondary objective of this study is to show noninferiority of the proportion of BDX-XL2malignant lung nodules routed to CT surveillance, preventing a potentially curative resection, under management by BDX-XL2 as compared to contemporaneous group not managed by BDX-XL2.
2. An additional secondary objective is to show noninferiority of the proportion of BDX-XL2 potentially curative resections of malignant nodules under management by BDX-XL2 as compared to a contemporaneous group not managed by BDX-XL2.

2.3 Exploratory Objectives

1. To evaluate and compare the standard of care risk assessment models.
2. To evaluate and compare the patterns of healthcare utilization between a group managed with BDX-XL2 and a contemporaneous group not managed by BDX-XL2.

3. Statistical Analysis

3.1 Study Population

There will be two defined study populations

i. Overall study population including all patients enrolled in the ORACLE registry

ii. Primary and Second Endpoint analysis population including all subjects enrolled in the ORACLE registry with definitive diagnoses of their identified nodules either by histopathology or radiology (stability or nodule resolved).

Formal statistical comparison of primary and secondary endpoints will be carried out with the Primary and Secondary Endpoint analysis population using appropriate statistical tests and methods prespecified in the formal statistical analysis plan (SAP). Statistical methods used for exploratory endpoints will be primarily descriptive in nature. Categorical variables will be summarized using frequency counts and percentages. Continuous variables will be summarized by total number of observations, mean, standard deviation, median and range. A formal SAP will be developed and finalized prior to statistical analysis of any study data. It will fully specify all statistical analyses to be performed along with the statistical methods to be used.

3.2 Sample Size Considerations

A survey of physicians in clinical practice indicates that nearly all patients eligible for this trial are likely to be directed to an invasive procedure under management without BDX-XL2. Prior data indicate that the rate of invasive procedures in patients with benign nodules under nodule management with BDX-XL2 is 52%. It would therefore be necessary to study 23 subjects in the trial and data from 23 contemporaneous subjects in the control group to be able to reject the null hypothesis that the rates of unnecessary invasive procedures are equal with 90% power and type I error $\alpha=0.05$, two-sided.

As the survey of physicians in clinical practice indicates that nearly all patients eligible for this trial are likely to be directed to an invasive procedure under management without BDX-XL2, the proportion of patients with malignant nodules directed to CT surveillance is very small. Prior data indicate that the proportion of

CONFIDENTIAL AND PROPRIETARY INFORMATION

The information contained within this document may not be used, divulged, published, or otherwise disclosed without prior written consent of Biodesix.

patients with malignant nodules with a Likely Benign test result who could be sent to CT surveillance under management with BDX-XL2 is 4%. For a noninferiority margin of 10% relative to management without BDX-XL2, it would be necessary to study 453 subjects in the trial and data from 453 contemporaneous subjects in the control group to be able to reject the null hypothesis of lack of noninferiority with the 10% margin with 80% power and type 1 error α=0.05, one-sided.

To examine primary and secondary endpoints allowing for loss to follow up, the study will enroll 500 subjects.

4. Study Plan

4.1 Registry Design

The ORACLE Registry study is a multicenter, observational research registry to evaluate the impact of the BDX-XL2 integrated classifier when used in the clinical management of a recently identified lung nodule with a low to moderate risk of cancer. Participation in the ORACLE Registry is a mandated requirement for coverage of the BDX-XL2 test for Medicare beneficiaries, however participation should be offered to all patients in the BDX-XL2 intended use population being treated by CTR-certified physicians for whom the BDX-XL2 test is being requested. For each patient participating in the registry, clinical information depicting the management of their lung nodule will be collected at baseline/enrollment and subsequent time points thereafter, until a study defined endpoint is met. Clinical information relevant to safety events (as defined by the registry protocol) will be collected as required throughout the duration of the registry.

A patient's decision to participate, or not participate in the BDX-XL2 Registry study should not influence or impact the clinical treatment they receive for their lung nodule of concern.

4.1.1 Description of Participating Sites

Biodesix will select qualified pulmonary practices as study sites. Site investigators will be evaluated for appropriate education, training and licensure, as well as relevant clinical and research experience. Registry sites will be evaluated for appropriate patient population and suitability of facilities and staff. Investigator and site evaluation will be performed by Biodesix or a designated representative.

Participating registry sites are required to obtain regulatory approval to conduct the study from a certified IRB or Ethics Committee. Regulatory approval must be obtained prior to any study-related subject activities and maintained throughout the duration of the study site's participation in the ORACLE Registry study.

Documentation of investigator and site credentials, approvals, training and qualifications will be collected and maintained by Biodesix. Additional documentation of policies and processes may be collected.

4.1.2 Overview of Data Collection

Except for BDX-XL2 assay results, all data collected for the ORACLE Registry will be entered by the site investigator (or qualified delegate) into the registry database through electronic, web-based case report forms (CRF). Data collected for study subjects includes specified demographics, clinical history, diagnostic procedures and results, clinical management plans, disposition and safety events. Study subject data will be collected during the initial patient enrollment and retrospectively at subsequent registry timepoints (every 6 months) until an endpoint has been met.

CONFIDENTIAL AND PROPRIETARY INFORMATION

The information contained within this document may not be used, divulged, published, or otherwise disclosed without prior written consent of Biodesix.

4.1.3 Overview of Biospecimen Collection

The BDX-XL2 assay will be ordered for participating patients during their registry enrollment timepoint. The site investigator will complete a Test Request Form (TRF) with the appropriate patient information and submit to Biodesix. A remote, qualified and trained home phlebotomy service will contact the patient and coordinate the collection of the BDX-XL2 sample. The phlebotomy service will collect, process, and ship the sample to Biodesix.

Approximately 2 - 3 weeks after sample collection, BDX-XL2 test results will be provided to the ordering physician via email, portal or fax.

Remnant samples will be retained by Biodesix and may be used for product improvement or product development purposes.

The BDX-XL2 tests performed for patients participating in the BDX-XL2 Registry are for use in routine clinical practice and as such will not be provided gratis as a condition of participation. All BDX-XL2 tests will be ordered as commercial requests and billed to Medicare/Medicaid and/or the patient's private insurance. For patients with private insurance, the insurance company will be billed for the BDX-XL2 test that the study doctor believes is medically necessary. Patients whose insurance is billed for the test may receive an Explanation of Benefits from their insurer, however costs related to BDX-XL2 that are not reimbursed by the insurance company will be covered by Biodesix. The patient will not have any out-of-pocket costs associated with the BDX-XL2 test.

4.1.4 Study Duration, Enrollment and Number of Sites

4.1.4.1 Duration of Study

Registry patients may be followed in the ORACLE Registry for up to 2 years from the baseline timepoint (Patient Enrollment). Patient participation in the ORACLE Registry will end when the patient reaches one of the following endpoints:

- Follow-up is no longer required:
  - Presumed benign diagnosis (i.e. nodule resolved or no growth of the lung nodule of concern after SOC CT surveillance)
  - Definitive diagnosis from histopathology
- Patient choice
- Patient lost to follow-up
- Patient expired

4.1.4.2 Total Number of Study Sites and Total Number of Subjects Projected

The study will be conducted at approximately 10 investigative sites in the United States. Investigative sites will be selected from qualified pulmonary practices.

It is expected that approximately 500 patients will be enrolled into the ORACLE Registry.

4.1.4.3 Study Population

Participation should be offered to all patients within the intended use population for whom the physician wishes to order the BDX-XL2 test.

CONFIDENTIAL AND PROPRIETARY INFORMATION

The information contained within this document may not be used, divulged, published, or otherwise disclosed without prior written consent of Biodesix.

4.1.5 INCLUSION CRITERIA

1. Patient has provided informed consent to participate in the registry and agrees to comply with all protocol requirements
2. Patient meets the criteria for the intended use population of the BDX-XL2 test:
    - Patient is $\geq$ 40 years of age at the time of the discovery of the lung nodule of concern
    - The maximal dimension of the patient's lung nodule of concern is $\geq$ 8mm and $\leq$ 30mm
    - The pre-test risk of cancer as determined by the Mayo risk prediction algorithm is 50% or less
3. The first CT scan identifying the lung nodule of concern was performed within 60 days of patient enrollment in the registry.

4.1.6 EXCLUSION CRITERIA

1. Nodule work-up before the time of patient enrollment indicating any prior attempted or completed diagnostic biopsy procedure
2. Current diagnosis of any active cancer
3. High risk per physician assessment.
4. Prior diagnosis of any cancer within 2 years of lung nodule detection, except for non-melanoma skin cancer
5. Concurrent participation in any unrelated clinical trial that may impact or alter the management of the patient's nodule of concern
6. Any illness or factor that will prevent compliance with follow-up as recommended

4.2 REGISTRY PROCEDURES

4.2.1 REGISTRY TIMEPOINTS

The BDX-XL2 Registry is designed to collect clinical data at timepoints throughout the duration of the management of a patient's lung nodule of concern. BDX-XL2 Registry Timepoints are:

- Patient Enrollment
- Post-Test Result Timepoint
- Follow-Up Timepoints
- Discontinuation/End of Study*

*** Discontinuation is not a stand-alone timepoint, but corresponds with the patient's final registry timepoint**

4.2.2 PATIENT ENROLLMENT

Patient Enrollment is the registry timepoint that confirms the patient's eligibility to participate in the ORACLE Registry, marks the patient's entry into the registry, and establishes the patient's baseline.

Patient Enrollment will occur within 60 days of the nodule of concern discovery and before any additional testing or diagnostic procedures on the nodule of concern. It is at this timepoint that the BDX-XL2 test is ordered.

The CT scan discovering the nodule of concern will be used as the patient's Baseline CT and source of Patient Enrollment data. This CT scan may not have been performed more than 60 days prior to Patient

CONFIDENTIAL AND PROPRIETARY INFORMATION

The information contained within this document may not be used, divulged, published, or otherwise disclosed without prior written consent of Biodesix.

Enrollment into the ORACLE Registry study. If an additional CT scan is performed expressly for the purpose of improved radiologic quality (not diagnostic follow-up) between new nodule discovery and Patient Enrollment, the repeat CT scan may be used as the patient's Baseline CT. The repeat CT scan will not alter the timing of the Patient Enrollment, which must still occur within 60 days of new nodule discovery.

Activities of Patient Enrollment timepoint are:

- Informed consent process will be completed and documented fully in the patient's source records.
- The site investigator will capture baseline patient data (to include demography, consenting information, eligibility information*, cancer history, smoking history and CT results for the lung nodule of concern) in the Registry's electronic CRFs.
- The site investigator will request the BDX-XL2 test by completing a BDX-XL2 TRF with the appropriate patient information and submit to Biodesix.

  *** Using the baseline patient data entered into the Registry eCRFs, the sponsor will confirm patient eligibility by calculating pCA with the Mayo risk prediction algorithm. The site investigator will be notified as any ineligible patients are identified.**

4.2.3 Post-Test Result Visit

The Post-Test Result Timepoint is a single registry timepoint that occurs when a BDX-XL2 test result is reviewed and the management plan for a patient's lung nodule is established.

Site investigators will be notified when BDX-XL2 test results for their patients are available (approximately 2-3 weeks after sample collection). After review and consideration of the patient's BDX-XL2 test result, the site investigator will determine their recommendations for clinical next steps. This recommendation will be recorded and discussed with the patient. Following discussion with the patient, the shared decision on a management plan will also be recorded.

The options for clinical next steps will be noted from the options provided:

- Continued CT Surveillance
- PET Scan
- Biopsy
- Surgery
- Other

It is anticipated that further evaluation of the nodule of concern awaits the BDX-XL2 test result. However, the patient will be queried and results of any CT scan, PET scan, biopsy or surgery (specific to the patient's lung nodule) since the patient's enrollment into the ORACLE Registry will be collected and evaluated.

NOTE: if a definitive diagnosis for the lung nodule of concern is available at this timepoint, no additional follow-up for the registry will occur and Discontinuation data will be collected and entered into the Registry's electronic CRFs. The site investigator will also assess if a safety event has occurred. (See section 7).

The site investigator will enter patient data collected during the Post Test Report Timepoint (to include management plan recommendations, shared decision for management plan and any radiology or procedure results) in the Registry's electronic CRFs. Safety Event and Discontinuation data will be entered as required.

CONFIDENTIAL AND PROPRIETARY INFORMATION

The information contained within this document may not be used, divulged, published, or otherwise disclosed without prior written consent of Biodesix.

Figure 1. Patient Enrollment & Post-Test Result

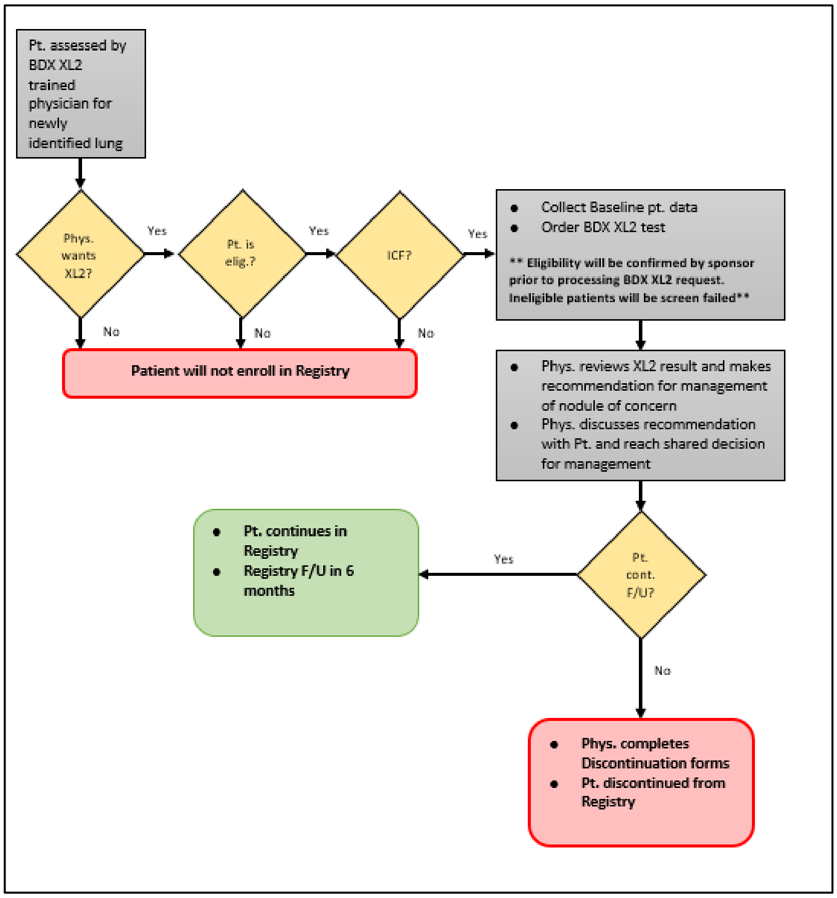

4.2.4 FOLLOW-UP TIMEPOINTS

Follow-Up Timepoints will occur in 6-month intervals (up to 24-months) following the Post-Test Report Timepoint until the patient has met a registry endpoint.

The purpose of the Follow-Up Timepoints is to retrospectively collect clinical data regarding the management of a patient’s nodule of concern since the previous registry timepoint.

Results of any CT scan, PET scan, biopsy or surgery (specific to the patient’s lung nodule) since the previous registry timepoint will be collected and evaluated.

NOTE: if a definitive diagnosis for the lung nodule of concern is available at this timepoint, no additional follow-up for the registry will occur and Discontinuation data will be collected and entered into the Registry’s electronic CRFs. The site investigator will also assess if a safety event has occurred. (See section 8)

The site investigator will enter patient data collected during Follow-Up Timepoint in the Registry’s electronic CRFs. Safety Event and Discontinuation data will be entered as required.

CONFIDENTIAL AND PROPRIETARY INFORMATION

The information contained within this document may not be used, divulged, published, or otherwise disclosed without prior written consent of Biodesix.

Figure 2. Follow-Up Timepoints

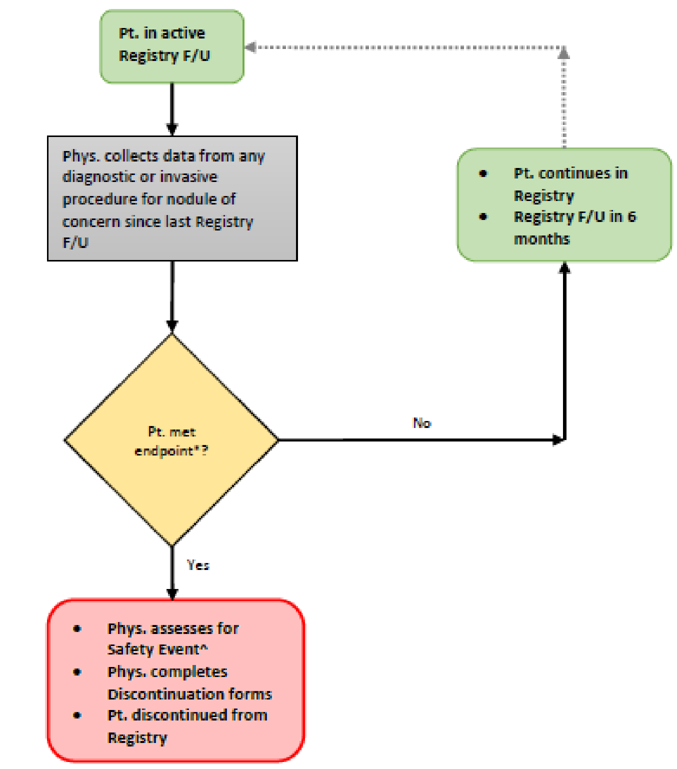

* Endpoint:

- Follow-Up no longer required:
  - Definitive diagnosis from histopath.
  - Radiologic diagnosis (radiologic stability or nodule resolved)
- Patient withdrew consent
- Patient is Lost-To-Follow-Up
- Patient expired

^ Safety Event occurs when <u>all 3</u> criteria are met:

- BDX XL2 result is <u>98% Likely Benign</u>
- Patient was followed primarily by <u>CT Surveillance</u>
- Subsequent malignant diagnosis for nodule of concern by histology/pathology

4.2.5 Discontinuation

Discontinuation is not a stand-alone registry timepoint but corresponds with the timepoint marking the end of a patient's participation in the ORACLE Registry study.

The purpose of the Discontinuation timepoint is to collect data regarding the patient's registry endpoint. Registry endpoints are:

- Follow-up is no longer required:
  - Presumed benign diagnosis (i.e. nodule resolved or no growth of the lung nodule of concern after SOC CT surveillance)
  - Definitive diagnosis from histopathology

CONFIDENTIAL AND PROPRIETARY INFORMATION

The information contained within this document may not be used, divulged, published, or otherwise disclosed without prior written consent of Biodesix.

- Patient choice
- Patient lost to follow-up
- Patient expired

Table 2. Registry Procedures

	Patient Enrollment	Post-Test Result	Follow-Up (6, 12, 18, 24 months)
Informed Consent Process	X		
Subject Eligibility	X		
Baseline Patient Information	X		
Baseline Clinical Information	X		
Baseline CT and Procedure Information	X		
Plasma Sample Collection	X		
Management Plan - Recommendation		X	
Management Plan – Shared Decision		X	
Invasive or Diagnostic Procedures since previous Registry timepoint		X	X
Discontinuation		X[1]	X[1]
Safety Report		X[2]	X[2]

[1] May be completed as required at any visit following BDX-XL2 test result

[2] Only completed when subject meets Safety Event criteria as defined in protocol section 12

4.3 Medical Data Collection Procedures

4.3.1 Data Collection

With the exception of BDX-XL2 test results, clinical information collected about and from registry patients during medical encounters with site investigators will be the source of the ORACLE Registry data. ORACLE Registry patient data may be extracted from procedure reports, test results, physical exams or historical medical records. Registry site investigators are responsible for maintaining adequate source documentation (i.e. EMR, medical chart, study source chart) for all participating registry patients. Registry site investigators are also responsible for the timely entry of patient data into the Registry electronic CRFs.

4.3.2 Protected Health Information Collected

Certain elements of Protected Health Information (PHI) are required for the commercial order of the BDX-XL2 test and will be collected for all participating patients. When ordering the BDX-XL2 test during patient enrollment, the site investigator will enter the following PHI elements onto the Test Request Form (TRF).

- Patient Name (First, Last, Middle Initial)
- Patient Address (Street address, City, State, Zip)
- Patient Phone Number (Primary, Secondary)
- Patient Date of Birth
- Patient Medical ID Number
- Patient Billing Information (Private Insurance, Medicare, Medicaid, Patient Pay, Account Bill)
- Patient Insurance Identification Number (and group number if applicable)

CONFIDENTIAL AND PROPRIETARY INFORMATION

The information contained within this document may not be used, divulged, published, or otherwise disclosed without prior written consent of Biodesix.

The only element of PHI that will be captured in the Registry electronic CRFs is the patient's date of birth.

5. Study Administration

Biodesix is the sponsor for the ORACLE Registry and will provide overall registry management and oversight of the ORACLE Registry. Prior to enrolling patients into the ORACLE Registry, investigators and sites selected to participate in the registry must complete the following activities:

- Registry site investigators must be selected for participation in the CTR and complete CTR certification training.
- Registry site investigators and relevant staff must participate in ORACLE Registry initiation training. A designated representative of Biodesix will conduct an onsite Site Initiation Visit with each participating site. (See section 7.1.2.)
- Participating investigators and sites must obtain regulatory approval to conduct the registry from an approved IRB or Ethics Committee. Quorum IRB will be utilized as the ORACLE Registry central IRB and available to registry sites not required to use their local IRB.
- Registry sites must provide all required regulatory essential documents to Biodesix. (See section 7.1.5.)

Upon meeting all start-up requirements, Biodesix will provide sites with written approval to begin patient enrollment into the ORACLE Registry.

5.1 Study Oversight

Biodesix is the sponsor for this clinical registry. Biodesix, in addition to contracted delegates, will provide oversight for the ORACLE Registry through site management and study monitoring.

5.1.1 Site Qualification Visits

Site Qualification Visits (SQV) will be conducted for all clinical sites and investigators being considered for participation in the ORACLE Registry. The purpose of a SQV is to evaluate site and investigator qualifications and suitability to successfully conduct the registry. Following a SQV, an evaluated site and investigator will be notified in writing of their selection/non-selection to participate in the ORACLE Registry.

5.1.2 Site Initiation Visits

Site Initiation Visits (SIV) will be conducted to formally train Registry sites and confirm preparedness to conduct the Oracle Registry. SIVs will be scheduled following execution of the site's Clinical Trial Agreement (CTA) and will be conducted at the site's location. As the SIV is the formal Registry training event, participation by the site investigator and relevant site research staff is required. Physician training conducted during the SIV will constitute certification.

5.1.3 Routine Monitoring Visits

Routine Monitoring Visits (RMV) will be conducted on-site at a frequency and duration determined by Registry needs. The purpose of monitoring, as required by federal regulations, is to:

- ensure all registry patients have been consented to participate in the Registry in accordance with regulatory guidelines
- evaluate the progress of the Registry
- verify the accuracy and completeness of captured patient data

CONFIDENTIAL AND PROPRIETARY INFORMATION

The information contained within this document may not be used, divulged, published, or otherwise disclosed without prior written consent of Biodesix.

- assure that all protocol requirements, applicable FDA/Health/GCP regulations, and investigator obligations are fulfilled
- assess or resolve any inconsistencies in the registry records and documentation
- ensure each site's regulatory files and essential documents are current, complete and available

All patient data and corresponding portions of office, hospital, and laboratory records (source documents) for each participating patient will be monitored. Captured registry data must be up-to-date and available for each monitoring visit. The site investigator and appropriate personnel must be available during the monitoring visits.

5.1.4 Close Out Visits

Close Out Visits will be conducted on-site following completion of all enrolled patients at a Registry site. COVs are the formal completion of a site's participation in the study.

5.2 Regulatory Oversight

As sponsor for the ORACLE Registry, Biodesix will maintain a central Trial Master File (TMF) to house all relevant registry documentation, to include:

- Finalized copies of registry guidance documents (original, amendments, addendums)
  - Protocol
  - Informed Consent
  - Registry conduct, guidance and training materials
- Program-level IRB submissions, approvals and correspondence
- Program-level site correspondence
- BDX-XL2 details
- Sponsor credentials
- Safety Data
- Other information as appropriate

Each participating registry site will maintain an Investigator Site File (ISF) to house all relevant Registry and site-specific documentation, to include:

- Finalized copies of registry guidance documents (original, amendments, addendums)
  - Protocol
  - Site-specific Informed Consent
  - Registry conduct, guidance and training materials
- Site-specific IRB submissions, approvals and correspondence
- Program and site-specific correspondence
- Registry management tools
  - Screening/Enrollment Log
  - Subject Visit Log
- BDX-XL2 details
- Site credentials and training materials
  - CVs and licenses
  - Delegation of Responsibilities Log
  - Registry training documentation
- Safety Data

CONFIDENTIAL AND PROPRIETARY INFORMATION

The information contained within this document may not be used, divulged, published, or otherwise disclosed without prior written consent of Biodesix.

- Other information as appropriate

For each participating registry site, Biodesix will also maintain individual Site Master Files (SMF) to house all relevant site-specific documentation. The SMFs (maintained by Biodesix) will be identical in content to their corresponding ISFs (maintained by the Registry sites).

6. Data Handling and Quality Assurance

6.1 Electronic Systems

All data collected for patients enrolled into the ORACLE Registry will be entered directly into the Registry database by site investigators or appropriately trained registry site delegates. Data will be captured in electronic CRFs housed on a web-based electronic data capture system (EDC). The EDC utilized for the ORACLE Registry is a secure, access-controlled, validated data collection system.

6.2 Confidentiality of Subjects

Patient PHI is collected for BDX-XL2 commercial billing purposes only and will not be used for any research or marketing purposes.

As patients are enrolled into the Registry, they will be assigned a unique registry number. Patient data will be identified by the unique registry number only.

6.3 Biospecimen Collection and Management

A mobile phlebotomy service will be utilized to collect all ordered BDX-XL2 test specimens. Following a BDX-XL2 test order, the mobile phlebotomy service will contact the patient directly and schedule the sample collection at a time and location that is convenient to the patient.

Remnant samples will be retained by Biodesix and may be used for product improvement or new product development purposes.

6.4 Reporting Results to Subjects

Registry patients will receive the results of their BDX-XL2 test from the ordering physician during the Post Test Result Timepoint. Analyses and results of the ORACLE Registry will not be reported to registry patients.

7. Regulatory and Ethical Considerations

7.1 Risk Assessment

As patient PHI will be used for the commercial order of the BDX-XL2 test and may be viewed by Biodesix staff during monitoring visits, the primary risk of participation in the ORACLE Registry is potential breach of privacy and confidentiality. The risk is no greater than minimal as all Biodesix employees involved in the ORACLE registry are required to complete annual training on HIPAA requirements and practices.

7.2 Potential Benefits of Participation

Participation in the ORACLE Registry may benefit patients directly through the results of their BDX-XL2 test. Participation will also contribute to the continued confirmation that the BDX-XL2 test performs as expected and influences nodule management decisions, which may lead to expanded use and coverage by healthcare insurances.

CONFIDENTIAL AND PROPRIETARY INFORMATION

The information contained within this document may not be used, divulged, published, or otherwise disclosed without prior written consent of Biodesix.

7.3 Recruitment Strategy

Eligible ORACLE Registry participants will be identified from participating site investigators' practice populations.

7.4 Informed Consent

Participation in the ORACLE Registry is a mandated requirement for coverage of the BDX-XL2 test for Medicare beneficiaries, however participation should be offered to all patients in the BDX-XL2 Registry study intended use population being treated by physicians for whom the BDX-XL2 test is being requested.

The informed consenting process will be completed in compliance with ICH GCP 4.8 (Informed Consent of Trial Subjects)

- The most current IRB-approved informed consent document (ICF) is to be presented to prospective registry patients during the consenting process.
- Patients must be provided adequate time to review the ICF and be given the opportunity to request additional information or clarification about the registry information contained in the ICF.
- No registry procedures may be performed prior to the completion of the consenting process.
- The ICF must be executed fully by the patient and appropriate site staff to document the patient's consent to participate in the registry and agreement to adhere to the requirements of the registry. A copy of the fully executed ICF will be provided to the patient.
- The consenting process will be documented in the patient's source records by the site staff.
- If important new information becomes available, the ICF will be revised to include the new information and submitted to IRB for approval. The consenting process will be repeated for all active/previously consented registry patients using the IRB-approved, revised ICF.

7.5 Payments to Patients

Registry patients will not be paid to participate in the ORACLE Registry. The IRB must review both the amount and method of payment to patients to ensure that neither presents an undue influence on the registry patients. Any reimbursement offered or provided to registry patients must be included in the informed consent.

7.6 Confidentiality

All data and records generated during this registry will be kept confidential in accordance with Institutional policies and HIPAA. Site investigators and other site personnel will not use such data and records for any purpose other than conducting the study.

8. Safety Management

8.1 Clinical Adverse Events

The only adverse events (AE) anticipated in this registry are those commonly associated with venipuncture. Patients may experience localized redness, tenderness, swelling or bruising at the BDX-XL2 sample collection site. Some patients may experience dizziness or lightheadedness during the BDX-XL2 sample collection. All adverse events should be managed as per local guidelines by the appropriate registry site clinical staff.

CONFIDENTIAL AND PROPRIETARY INFORMATION

The information contained within this document may not be used, divulged, published, or otherwise disclosed without prior written consent of Biodesix.

As registry procedures are not greater than minimal risk, serious adverse events (SAE) are not expected. AEs and SAEs will not be recorded for the ORACLE Registry.

Unanticipated problems involving risks to patients and others will be monitored throughout the registry.

8.2 Safety Events

For the purposes of the ORACLE Registry, a safety event is any occurrence that meets **all 3** of the following criteria:

- The BDX-XL2 result reports as Likely Benign at a 98% post-test probability
- The patient's lung nodule of concern was managed by CT surveillance for at least 6 months following BDX-XL2 results
- The patient's lung nodule of concern was diagnosed as malignant by histopathology

8.3 Safety Event Reporting

Any suspected occurrence of a safety event (as per the study definition – section 7.2.) will be submitted to Biodesix for confirmation.

Biodesix contact for safety event confirmation or questions:

Russell Hudnall
Director of Clinical Operations
(206) 576 6388
russell.hudnall@biodesix.com

Any unanticipated problems related to the BDX-XL2 test or this registry that involves risks to patients or others will be communicated to participating registry sites and reported to the appropriate regulatory organizations.

9. Publication Plan

- Primary Endpoint Analyses
- Final Primary and Secondary Endpoint Analysis
- Final Report

10. References

1. Gould, M.K., et al., *Recent Trends in the Identification of Incidental Pulmonary Nodules.* Am J Respir Crit Care Med, 2015. **192**(10): p. 1208-14.
2. Nichole T. Tanner, M., MSCR; Jyoti Aggarwal, MS; Kenneth Fang, MD; Paul Kearney, PhD; Michael K. Gould, MD, MS; Gregory Diette, MD, MHS; Anil Vachani, MD, MS; Gerard A. Silvestri, MD, MS, *Management of Pulmonary Nodules by Community Pulmonologists A Multicenter Observational Study.* CHEST Journal, 2015: p. 1-24.
3. Kearney, P., et al., *An integrated risk predictor for pulmonary nodules.* PLoS One, 2017. **12**(5): p. e0177635.
4. Silvestri, G.A., et al., *Assessment of Plasma Proteomics Biomarker's Ability to Distinguish Benign From Malignant Lung Nodules: Results of the PANOPTIC (Pulmonary Nodule Plasma Proteomic Classifier) Trial.* Chest 2018.
5. Christine M. Micheel, S.J.N., and Gilbert S. Omenn, Editors, *Evolution of Translational Omics Lessons Learned and the Path Forward Committee: Committee on the Review of Omics-Based*

CONFIDENTIAL AND PROPRIETARY INFORMATION

The information contained within this document may not be used, divulged, published, or otherwise disclosed without prior written consent of Biodesix.

Tests for Predicting Patient Outcomes in Clinical Trials. Institute of Medicine of the National Academies: p. 1-274.

6. Balekian, A.A., et al., *Accuracy of clinicians and models for estimating the probability that a pulmonary nodule is malignant.* Ann Am Thorac Soc, 2013. **10**(6): p. 629-35.
7. E.S., S.S.J.S.M.D.I.D.M.S.C.D.E., *The Probability of Malignancy in Solitary Pulmonary Nodules: Application to Small Radiologically Indeterminate Nodules.* Archives of Internal Medicine, 1997(157): p. 849-855.
8. Gould, M.K., et al., *A clinical model to estimate the pretest probability of lung cancer in patients with solitary pulmonary nodules.* Chest, 2007. **131**(2): p. 383-8.
9. McWilliams, A., et al., *Probability of cancer in pulmonary nodules detected on first screening CT.* N Engl J Med, 2013. **369**(10): p. 910-9.
10. G. J. Herder, H.v.T., R. P. Golding, P. J. Kostense, E. F. Comans, E. F. Smit and O. S. Hoekstra, *Clinical Prediction Model to Characterize Pulmonary Nodules: Validation and Added Value of 18F-Fluorodeoxyglucose Positron Emission Tomography.* CHEST Journal, 2005. **128**(4): p. 2490-2496.

CONFIDENTIAL AND PROPRIETARY INFORMATION

The information contained within this document may not be used, divulged, published, or otherwise disclosed without prior written consent of Biodesix.

Appendix A – Control Group Sub-Study for ORACLE Registry

1.0 Introduction - Background and Rationale

Biodesix is conducting the ORACLE Registry; a multicenter, observational research registry study evaluate the impact of the BDX-XL2 integrated classifier on lung nodule management when used in the clinical management of recently identified lung nodules assessed to have a lower risk of cancer.

The Control Group Sub-Study has been designed to establish a contemporaneous control group for comparison with patients enrolled into the ORACLE Registry.

2.0 Objective

To establish a control group for comparison with the ORACLE Registry subject population to facilitate the ORACLE Registry objectives.

3.0 Selection of Patients

Sites/Investigators participating in the Control Group Sub-Study will identify patients from within their own practice in a systematic way (continuous or random sampling). Source documentation representative of the entirety of the management of the patient's lung nodule of concern must be available in the patient's medical records.

3.1 Inclusion Criteria:

- Patient underwent diagnostic evaluation, and subsequent management for a newly discovered lung Nodule of Concern identified by CT scan
- Patient was $\geq$ 40 years of age at the time of the Nodule of Concern discovery
- The maximal dimension of the lung Nodule of Concern was $\geq$ 8mm and $\leq$ 30mm at discovery
- The pre-test risk of cancer as determined by the Mayo risk prediction algorithm is 50% or less
- Evaluation and management of the Nodule of Concern began no earlier than June 2015 and ended no later than September 2018

3.2 Exclusion Criteria:

- Concurrent diagnosis of any active cancer at the time of lung nodule discovery
- Prior diagnosis of any cancer within 2 years of lung nodule detection, except for non-melanoma skin cancer

4.0 Study Methods

4.1 Selection of charts to be reviewed:

The patients included in this retrospective chart review will be selected from within the participating site investigator's practice population in a systematic way (continuous or random sampling). The site investigator will identify patients meeting inclusion and exclusion criteria. (See Section 3.0).

Data to be collected is representative of the entirety of the management of the patient's lung nodule of concern and will collect the same data points as that of the prospective registry patients:

4.2 Source of records to be reviewed:

CONFIDENTIAL AND PROPRIETARY INFORMATION

The information contained within this document may not be used, divulged, published, or otherwise disclosed without prior written consent of Biodesix.

Individual patient medical records maintained by the site investigator's practice will serve as the data source for the chart review. The source records may be paper or electronic (EMR), or a combination of both. Documentation representative of the entirety of the management of the patient's lung nodule of concern must be available in the patient's medical records.

The site investigator (or appropriate authorized delegate) will review the medical records maintained by the practice for the identified patients and capture required patient data on case report forms.

4.3 Identification of reviewed charts:

Personal identifiers and protected health information for patients included in the chart review will not be collected for the analysis database. A unique Subject Number will be assigned to each patient for whom data is collected. A separate Coded Patient Identifier List will be maintained by the site investigator to link data captured in case report forms to the source medical records.

5.0 Confidentiality of data

5.1 Data Storage:

The data collected for the Control Group Sub-Study will be captured on electronic case report forms housed on a validated electronic data capture system/database. Access to the study EDC is password-controlled for all study site and sponsor staff.

The study site's source medical records will be maintained and secured in compliance with the site's local policies.

5.2 Data Access:

The study sponsor will have access to the anonymized patient data housed in the electronic data capture system. Representatives of the study sponsor will require unblinded access to the patients' source medical records for purposes of source data verification.

6.0 Consent:

The waiver of consent is justified by the following:

- The research involves no more than minimal risk to the subject
- The waiver will not adversely affect the rights and welfare of the subjects;
- The research could not practicably be carried out without the waiver or alteration

7.0 Risks and Benefits:

7.1 Risks:

There are no health risks to patients associated with this retrospective chart review study. While all efforts will be made to maintain the security of the collected patient data, a risk of confidentiality breach cannot be eliminated.

7.2 Benefits:

The patient's whose data will be collected are not likely to receive any benefit from this study; however, society and investigators will benefit from the knowledge gained.

8.0 Statistical Considerations

Data for approximately 200 – 500 patients will be collected for this sub-study population. All sites participating in the ORACLE Registry will be required to participate in the Control Group Sub-Study.

CONFIDENTIAL AND PROPRIETARY INFORMATION

The information contained within this document may not be used, divulged, published, or otherwise disclosed without prior written consent of Biodesix.

Data collected from this retrospective study will be used to form a contemporaneous control group for the analysis of patient data collected from the BDX-XL2 Registry.

CONFIDENTIAL AND PROPRIETARY INFORMATION

The information contained within this document may not be used, divulged, published, or otherwise disclosed without prior written consent of Biodesix.

Appendix B – Results of the PANOPTIC Trial

ARTICLE IN PRESS

[Original Research]

CHEST

Assessment of Plasma Proteomics Biomarker's Ability to Distinguish Benign From Malignant Lung Nodules

Results of the PANOPTIC (Pulmonary Nodule Plasma Proteomic Classifier) Trial

*Gerard A. Silvestri, MD; Nichole T. Tanner, MD; Paul Kearney, PhD; Anil Vachani, MD; Pierre P. Massion, MD; Alexander Porter, MD; Steven C. Springmeyer, MD; Kenneth C. Fang, MD; David Midthun, MD; Peter J. Mazzone, MD, MPH; for the PANOPTIC Trial Team**

BACKGROUND: Lung nodules are a diagnostic challenge, with an estimated yearly incidence of 1.6 million in the United States. This study evaluated the accuracy of an integrated proteomic classifier in identifying benign nodules in patients with a pretest probability of cancer (pCA) ≤ 50%.

METHODS: A prospective, multicenter observational trial of 685 patients with 8- to 30-mm lung nodules was conducted. Multiple reaction monitoring mass spectrometry was used to measure the relative abundance of two plasma proteins, LG3BP and C163A. Results were integrated with a clinical risk prediction model to identify likely benign nodules. Sensitivity, specificity, and negative predictive value were calculated. Estimates of potential changes in invasive testing had the integrated classifier results been available and acted on were made.

RESULTS: A subgroup of 178 patients with a clinician-assessed pCA ≤ 50% had a 16% prevalence of lung cancer. The integrated classifier demonstrated a sensitivity of 97% (CI, 82-100), a specificity of 44% (CI, 36-52), and a negative predictive value of 98% (CI, 92-100) in distinguishing benign from malignant nodules. The classifier performed better than PET, validated lung nodule risk models, and physician cancer probability estimates ($P < .001$). If the integrated classifier results were used to direct care, 40% fewer procedures would be performed on benign nodules, and 3% of malignant nodules would be misclassified.

CONCLUSIONS: When used in patients with lung nodules with a pCA ≤ 50%, the integrated classifier accurately identifies benign lung nodules with good performance characteristics. If used in clinical practice, invasive procedures could be reduced by diverting benign nodules to surveillance.

TRIAL REGISTRY: ClinicalTrials.gov; No.: NCT01752114; URL: www.clinicaltrials.gov).

CHEST 2018; ■(■):■-■

KEY WORDS: biomarker; diagnosis; lung cancer; proteomics; pulmonary nodules; risk models

ABBREVIATIONS: AUC = area under the receiver-operating characteristic curve; NPV = negative predictive value; pCA = probability of cancer; TTNA = transthoracic needle biopsy; VA = Veterans Affairs

AFFILIATIONS: From the Thoracic Oncology Research Group Division of Pulmonary and Critical Care Medicine (Drs Silvestri and Tanner), Medical University of South Carolina, Charleston, SC; Health Equity and Rural Outreach Innovation Center (Dr Tanner), Ralph H. Johnson Veterans Affairs Hospital, Charleston, SC; Integrated Diagnostics (Drs Kearney, Porter, Springmeyer, and Fang), Seattle, WA; Division of Pulmonary, Allergy, and Critical Care Medicine (Dr Vachani), Penn Lung Center, Perelman School of Medicine, University of Pennsylvania, Philadelphia, PA; Thoracic Program (Dr Massion),

CONFIDENTIAL AND PROPRIETARY INFORMATION

The information contained within this document may not be used, divulged, published, or otherwise disclosed without prior written consent of Biodesix.

Chest CT imaging has increasingly been used for a myriad of medical presentations such that the estimated incidence of pulmonary nodules in the United States is 1.6 million per year.[1] Add to that the findings of the National Lung Screening Trial, which showed low-dose chest CT imaging led to a nodule detection rate of 25% on the prevalence screen, the evaluation and management of pulmonary nodules are certain to be an increasingly common issue needing to be addressed.[2]

The approach to a pulmonary nodule begins with estimating the probability of malignancy.[3] Depending on probability of cancer (pCA), patients are managed with either CT surveillance, additional diagnostic testing (including PET, CT-guided transthoracic needle biopsy [TTNA], and/or bronchoscopy), or definitive therapy with surgical excision. All approaches should account for patient health status and preferences. Although physicians are skilled at predicting malignancy in pulmonary nodules, they may not follow guideline-based care when managing patients with differing risks.[4,5]

We previously developed a 13-protein blood test to differentiate benign from malignant lung nodules using multiple reaction monitoring mass spectrometry.[6] The proteomic classifier was more accurate than a four-parameter clinical model, independent of patient age, tobacco use, nodule size, and the presence of COPD.[7] The test was further refined to improve performance for lower risk nodules in which the diagnostic dilemma between surveillance and invasive procedures is most challenging. It was discovered that the accuracy of two of the proteins, LG3BP and C163A (independently linked to lung cancer and the inflammatory response to cancer),[8-10] could be optimized for evaluating lower risk nodules by integrating them with five clinical risk factors. LG3BP, in particular, has been previously studied in a network analysis implicating its overexpression to lung cancer pathways and associated transcription factors.[6] This integrated classifier was trained on an independent set of 222 prospectively collected plasma samples from patients with 8- to 20-mm lung nodules.[11] The current article is a second prospective, multicenter, observational clinical validation study with retrospective evaluation assessing the performance characteristics of this integrated classifier in patients presenting with undiagnosed low to moderate risk lung nodules.

Patients and Methods

Pulmonary Nodule Plasma Proteomic Classifier Study Design and Enrollment

The Pulmonary Nodule Plasma Proteomic Classifier (PANOPTIC) study is a prospective, multicenter, observational study with retrospective evaluation of the performance of the integrated classifier test comprising two proteins and five clinical risk factors. Physicians, study subjects, and laboratory and statistical personnel were blinded to the results of the test and clinical information. The blinding protocol was strictly followed, and the results of the test did not direct or influence patient care. Thirty-three sites (31 US and two Canadian) were included. All sites had institutional review board approval, and informed written consent was obtained from all eligible participants (e-Table 1).

Eligible patients were ≥ 40 years old with pulmonary nodules 8 to 30 mm in diameter presenting within 60 days of the baseline CT scan to a pulmonologist and/or a thoracic surgeon. Mediastinal lymphadenopathy was not an exclusion. Patients were ineligible if they had any of the following: undergone any attempt at a previous biopsy of the nodule in question; undergone a prior CT scan or PET/CT scan that had identified the lung nodule under consideration; a current or previous diagnosis of any cancer within 2 years of lung nodule detection (except for nonmelanoma skin cancer); or received any blood products within 30 days of study enrollment.

A power analysis was performed to assess the number of benign and malignant nodules required to perform a statistical test of significance for area under the receiver-operating characteristic curve (AUC) assuming an α of 0.05, power of 0.80, and an assumed performance with AUC of 0.70. The number necessary to have adequate power was 21 benign and 21 malignant nodules.

Data Collection

At enrollment and prior to subsequent testing, physicians (pulmonologists or thoracic surgeons to whom the patients had been referred) assessed nodule pretest pCA as 0% to 5%, 6% to 10%, 11% to 20%, and so forth. Physicians estimated the pretest probability at their own discretion. Patient demographic and nodule characteristics were collected at baseline and from subsequent CT imaging studies. Imaging studies were interpreted by site radiologists as part of usual care, and nodule characteristics (including location,

Vanderbilt-Ingram Comprehensive Cancer Center, Nashville, and Veterans Affairs, Tennessee Valley Healthcare System, Nashville Campus, Nashville, TN; Mayo Clinic (Dr Midthun), Rochester MN; and the Respiratory Institute (Dr Mazzone), Cleveland Clinic, Cleveland, OH.

Drs Silvestri and Tanner contributed equally to this article.

FUNDING/SUPPORT: Integrated Diagnostics provided funding for the study as well as laboratory and biostatistical support.

*A list of investigators and coordinators in the PANOPTIC (Pulmonary Nodule Plasma Proteomic Classifier) trial is provided in the supplementary material.

CORRESPONDENCE TO: Gerard A. Silvestri, MD, Medical University of South Carolina, 96 Jonathan Lucas St, Room 816 CSB, Charleston, SC 29425; e-mail: silvestri@musc.edu

Published by Elsevier Inc. under license from the American College of Chest Physicians. This is an open access article under the CC BY-NC-ND license (http://creativecommons.org/licenses/by-nc-nd/4.0/).

DOI: https://doi.org/10.1016/j.chest.2018.02.012

CONFIDENTIAL AND PROPRIETARY INFORMATION

The information contained within this document may not be used, divulged, published, or otherwise disclosed without prior written consent of Biodesix.

size, and edge characteristics) were collected from the reports by site study personnel. Blood samples were obtained and processed for storage and shipment. Data on subsequent procedures, including bronchoscopy, transthoracic needle biopsy, and surgery, were obtained and entered by the enrolling site until definitive diagnosis, nodule resolution, or at least 1 year of radiographic stability according to chest CT imaging. PET results were recorded as positive for standard uptake values ≥ 2.5. Pathology reports were collected from biopsy samples or surgical resection.

Plasma Proteomic Analysis

Proteomic analysis of two plasma proteins, LG3BP and C163A, was performed by using multiple reaction monitoring mass spectroscopy as previously described.[6,7] These results were integrated with the five clinical risk factors (age [in years], smoking status [never, current, or former], nodule diameter [largest diameter], edge characteristics [smooth, spiculated, or lobulated], and location]) to yield a posttest probability of a lung nodule being benign as previously defined in a separate prospective study (e-Fig 1, e-Table 2).[11]

Analysis of Integrated Classifier Performance

The performance of the integrated classifier was assessed by using standard metrics, including AUC, sensitivity, specificity, and negative predictive value (NPV). These metrics were also used to compare the performance of the integrated classifier vs other lung nodule cancer risk stratification methods, including physician assessment (pCA), clinical prediction models (Mayo and Veterans Affairs [VA]), and PET. Because the intended use of the integrated classifier is to identify benign lung nodules and reduce the number of invasive procedures, the performance of the test, and its comparators, was assessed by using the NPV as the primary metric. The threshold at which maximum NPV was observed in the discovery study was validated in the current study.[11] Furthermore, the NPV at this threshold in the current study is used to report the performance of the test.

Nodules were defined as benign for any of the following: (1) definitive pathologic diagnosis; (2) radiographic resolution; or (3) no evidence of growth according to CT scan over 1 year. Although the standard surveillance is 2 years of radiologic stability on chest CT imaging, for this analysis, 1 year of CT imaging stability was chosen based on a study observing lung nodules/masses by CT scan demonstrating no growth in nodules stable at 1 year on subsequent 2-year follow-up.[12] A malignant diagnosis was based on histopathologic findings. Those patients who were treated empirically with radiation and/or chemotherapy for a suspected malignancy without a confirmatory diagnosis were excluded from the analysis.

Assessment of Potential Impact of the Integrated Classifier

In the intended use cohort, those nodules with a "likely benign" integrated classifier result were stratified according to final diagnosis. Percentages of benign nodules correctly classified by using the integrated classifier and malignant nodules incorrectly classified by using the integrated classifier were calculated. Invasive procedure utilization directly following initial nodule detection was tabulated and stratified according to final diagnosis to assess the number and percentages of invasive testing that could have been avoided had the integrated classifier test been available and used for nodule management.

Data Analysis

Statistical analyses were performed by using MATLAB, version 8.3.0.532 (MathWorks), and MedCalc, version 16.4 (MedCalc Software bvba). A χ^2 of ANOVA testing was used to compare groups, and a P value ≤ .05 was considered significant. For comparison of cancer risk predictors, the McNemar statistical test was used at a fixed sensitivity or specificity.[13]

Results

Of 685 patients enrolled prospectively in the PANOPTIC study from November 2012 to December 2015, a total of 293 were excluded for the reasons listed in Figure 1, yielding 392 eligible for analysis. The demographic characteristics for the 392 patients are presented in e-Table 1. This study of the integrated classifier's performance focused on the subgroup of 178 patients having a lung nodule with a pCA ≤ 50%. No differences were observed in demographic or nodule characteristics between included and excluded (n = 234) patients (e-Table 3). Table 1 provides baseline patient demographic characteristics and radiographic parameters for nodules having a pCA ≤ 50%, stratified according to final histologic diagnosis. The prevalence of malignancy was 16%, with the majority being adenocarcinoma histology (59%). Both groups had significant smoking histories. Malignant nodules were significantly larger (16.5 vs 13.5 mm; $P < .01$).

The demographic characteristics, outcomes, and procedure utilization across groups of physician-assessed risk of malignancy are presented in e-Table 4. The risk of cancer increases with nodule size ($P < .001$). The use of serial imaging decreases, while the use of PET, biopsy, and surgery all increase, as the risk of malignancy increases ($P < .001$).

For all 392 eligible subjects, clinicians assigned a PCA ≤ 50% to the majority with an eventual benign diagnosis, with only 6% of subjects assigned to the very lowest risk group with a pCA of 0% to 5% (Fig 2). The performance of clinician assessments (pCA) was compared with that of the VA and Mayo Clinic lung nodule risk models.[14,15] The AUC for physician assignment of benign nodules into low to moderate risk (≤ 50%) or malignant nodules into moderate to high risk (> 50%) groups was 0.85, which was greater than the AUCs of 0.75 and 0.78 observed for the VA and Mayo models, respectively.[5]

Further analyses focused on the pCA ≤ 50% subgroup (n = 178). The integrated classifier revealed a sensitivity of 97% (CI, 82-100), a specificity of 44% (CI, 36-52), and a posttest probability of 98% (CI, 92-100) in distinguishing benign from malignant nodules. The integrated classifier demonstrated an AUC of 0.76,

CONFIDENTIAL AND PROPRIETARY INFORMATION

The information contained within this document may not be used, divulged, published, or otherwise disclosed without prior written consent of Biodesix.

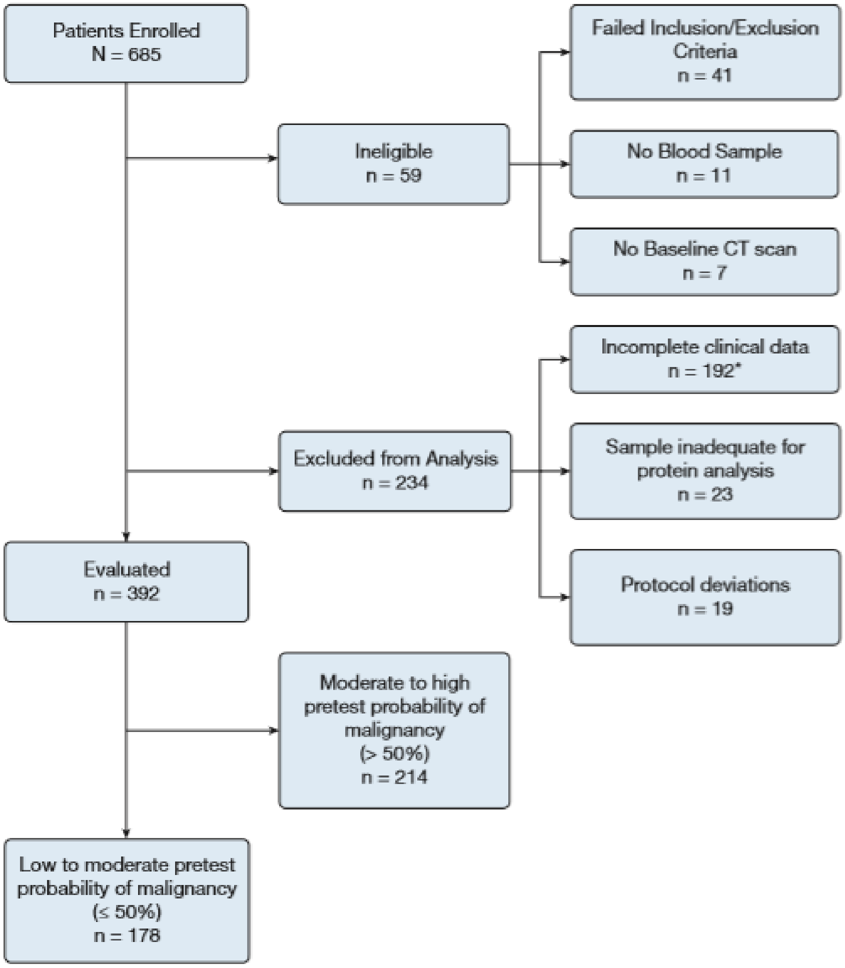

Figure 1 – *Eligibility of the Pulmonary Nodule Plasma Proteomic Classifier study patients for integrated classifier performance analysis in lung nodules according to the probability of malignancy. *Incomplete clinical data are broken down as follows: n = 9, no pretest probability provided; n = 48, no follow-up procedure documented; n = 88, no 1-year follow-up CT scan; n = 39, no follow-up after PET scan; n = 5, time between interval scans did not reach 1 year; and n = 3, biopsy performed without documentation of results.*

whereas the AUCs for physician pCA, the VA and Mayo models, and PET were 0.69, 0.60, 0.69, and 0.58, respectively (Fig 3). In e-Figure 2 and e-Table 5, the relative contribution of the component elements of the integrated classifier are presented. Using the McNemar test in which sensitivity was fixed at 90%, the integrated classifier performed significantly better than physician pCA, the VA and Mayo models, and PET ($P \leq .001$). Comparison to PET was restricted to the 75 subjects who had PET performed. Similar performance of the integrated classifier when stratified according to lung cancer subtype is shown in e-Table 6. The effects of the integrated classifier on adjusting pCA in the intended-use population stratified according to benign and malignant diagnoses are shown in e-Figure 3.

The potential impact of the integrated classifier was evaluated in two populations. For intended-use subjects, 66 of 178 were "likely benign" according to the integrated classifier. Of these, 65 had a benign nodule and 1 had a malignant lung nodule. Because there are 149 benign lung nodules and 29 malignant lung nodules in the study, 44% of benign lung nodules (65 of 149) were correctly labeled "likely benign," and 3% of malignant nodules

CONFIDENTIAL AND PROPRIETARY INFORMATION

The information contained within this document may not be used, divulged, published, or otherwise disclosed without prior written consent of Biodesix.

TABLE 1] Demographic Characteristics of the PANTOPIC Cohort With Lung Nodule pCA ≤ 50%

Characteristic	All Patients	Cancer	Benign	*P* Value
No. of patients	178	29	149	
Age, y	65.52 ± 1.55	66.05 ± 3.05	65.42 ± 1.76	.772
Sex				
Male	95 (53.37%)	12 (41.38%)	83 (55.70%)	.157
Female	83 (46.63%)	17 (58.62%)	66 (44.30%)	
Smoking history				
Status				.855
Never	42 (23.60%)	6 (20.69%)	36 (24.16%)	.725
Former	99 (55.62%)	16 (55.17%)	83 (55.70%)	.972
Current	37 (20.79%)	7 (24.14%)	30 (20.13%)	.665
Pack-year mean	43.56 ± 6.17	43.66 ± 11.73	43.54 ± 7.06	.989
Lung nodule				
Size	13.95 ± 0.76	16.48 ± 2.18	13.46 ± 0.78	.006
Nodule location				
Upper lobe		20 (68.97%)	70 (46.98%)	.128
Lower lobes		9 (31.03%)	79 (53.02%)	.123
Histology				
Benign nodule diagnosis				
Granuloma			9 (6.04%)	
Hamartoma			6 (4.03%)	
CT scan stable/resolution			116 (77.85%)	
Other			15 (10.07%)	
NA			3 (2.01%)	
Malignant nodule type				
Adenocarcinoma		17 (58.62%)		
Squamous cell		4 (13.79%)		
Large cell		0		
Mixed/nonspecified NSCLC		1 (3.45%)		
Small cell		2 (6.90%)		
Carcinoid		3 (10.34%)		
Other		2 (6.90%)		

Data are presented as mean ± SD unless otherwise indicated. NA = not available; NSCLC = non-small cell cancer lung cancer; PANOPTIC = Pulmonary Nodule Plasma Proteomic Classifier; pCA = probability of cancer.

(1 of 29) were incorrectly labeled "likely benign." In the 58 subjects who underwent an invasive procedure directly following the initial nodule detection, 35 nodules were benign and 23 were malignant. Of the 35 benign lung nodules, 14 (40%) were identified as "likely benign" by the integrated classifier, whereas of the 23 malignant lung nodules, 1 (4%) was incorrectly identified as "likely benign." The actual number of malignant nodules routed initially to surveillance was 13 of 29 (45%) in the intended-use subjects.

Table 2 details invasive diagnostic procedure utilization for nodules with pCA ≤ 50% stratified according to histologic diagnosis, as well as potential reduction of procedure had the integrated classifier been used in decision-making. Among surgeries performed as the sole diagnostic procedure, 7 of 20 (35%) were benign. Among biopsies performed as the only diagnostic procedure, 28 of 37 (76%) were benign. Fifty percent of patients who underwent both biopsy and surgical procedures received a benign diagnosis.

Discussion

This trial is the first clinical validation study evaluating the performance characteristics of an integrated blood

CONFIDENTIAL AND PROPRIETARY INFORMATION

The information contained within this document may not be used, divulged, published, or otherwise disclosed without prior written consent of Biodesix.

ARTICLE IN PRESS

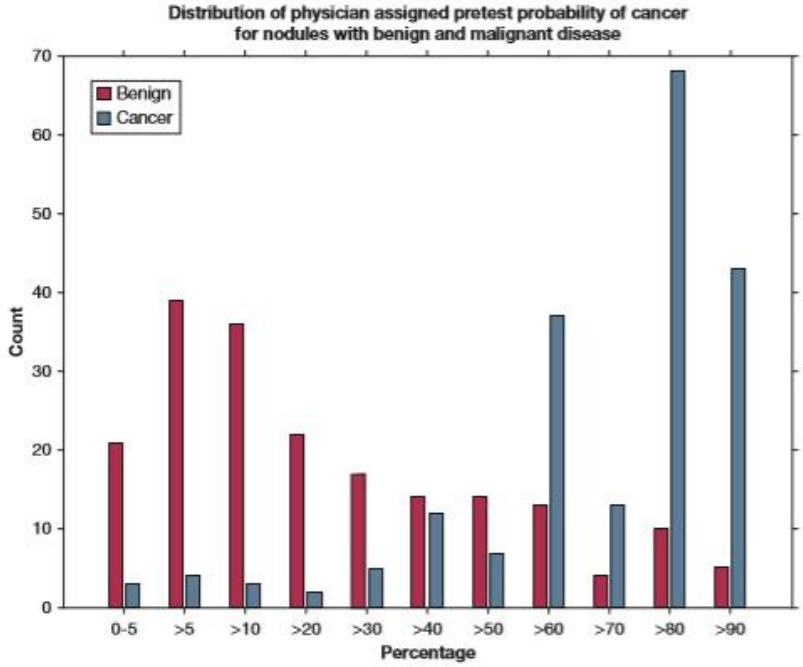

Figure 2 – *Distribution of physician-assigned pretest pCA for eligible Pulmonary Nodule Plasma Proteomic Classifier study patients (n = 392) by deciles. Shown are the physician-assigned pCA percentages for nodules with either a malignant (n = 197) or benign (n = 195) diagnosis. Note: the first two columns represent 5% pCA increments. pCA = probability of cancer.*

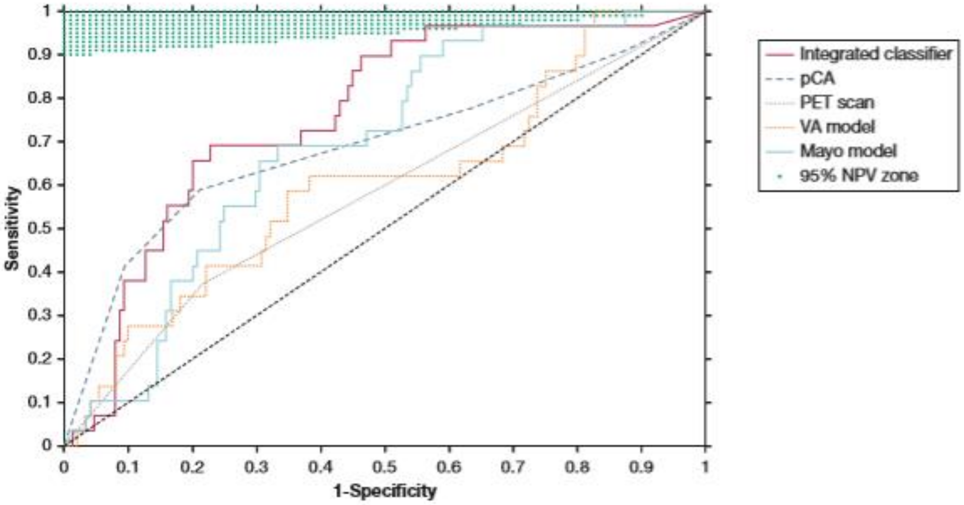

Figure 3 – *Comparison of the area under the receiver-operating characteristic curves of lung nodule malignancy risk assessment tools relative to the 95% NPV zone. Shown are the receiver-operating characteristic curves for subjects with lung nodules assigned a pCA ≤ 50% (n = 178) comparing the integrated classifier vs the physician-assigned pCA, PET, and the VA and Mayo cancer risk models. The shaded area indicates the ≥ 95% NPV diagnostic performance zone, which corresponds to the 5% cancer risk threshold specified in the CHEST guidelines for lung management. NPV = negative predictive value; VA = Veterans Affairs. See Figure 2 legend for expansion of other abbreviation.*

CONFIDENTIAL AND PROPRIETARY INFORMATION

The information contained within this document may not be used, divulged, published, or otherwise disclosed without prior written consent of Biodesix.

TABLE 2] Use of Invasive Diagnostic Procedures in the PANOPTIC Study Subgroup With Lung Nodules Having pCA ≤ 50%

Variable	No.	% of Category	No. of Procedures if Integrated Classifier Used	RRR	Misclassified Patient
All patients with invasive procedures	71				
Benign nodule patients	42	59	27	36%	1
Malignant nodule patients	29	41	28		
All patients with direct[a] invasive procedures	58				
Benign nodule patients	35	60	21	40%	1
Malignant nodule patients	23	40	22		
Patients with surgical procedures alone	20				
Benign nodule patient receiving surgery	7	35	5	29%	1
Malignant nodule patient receiving surgery	13	65	12		
Patients with biopsies alone	37				
Benign nodule patients receiving biopsy	28	76	16	43%	0
Malignant nodule patients receiving biopsy	9	24	9		
Patients with biopsies and surgical procedures	14				
Benign biopsy and surgery patients	7	50	6	14%	0
Malignant biopsy and surgery patients	7	50	7		

RRR = relative risk reduction. See Table 1 legend for expansion of other abbreviations.
[a]Direct denotes that the patient was not placed into CT surveillance but entered into a pathway that led directly to an invasive procedure.

proteomics classifier, comprising both proteins and clinical parameters, in distinguishing benign from malignant nodules. This large, multicenter observational study has several important findings. First, over the entire risk space, physicians perform very well; however, they are less accurate when the pCA is ≤ 50%. Second, when the probability of malignancy in a nodule is ≤ 50%, a "likely benign" integrated proteomic classifier result accurately identifies patients with benign nodules. This approach may enable physicians to re-categorize nodules from a strategy in which further testing is indicated to one in which CT surveillance is advisable. Thus, if incorporated into the current algorithm for managing nodules, the test may reduce patients' exposure to the morbidity and cost of avoidable invasive procedures. In addition, use of the test would lead to only a small percentage of patients with malignancy being misclassified as benign, potentially leading to an inappropriate surveillance strategy. These findings substantiate the need for a future clinical utility study to assess how clinical decision-making and use of invasive procedures are altered with knowledge of the results of this test in the right clinical setting.

When choosing a strategy for evaluating patients with lung nodules, clinicians should consider both the probability that the nodule is malignant and the advantages and disadvantages of management strategies. Serial surveillance has the advantage of being noninvasive and is recommend if the pCA is < 5%. However, in this study and another, < 10% of nodules ≥ 8 mm fell into this risk category.[4] In the latter study, all patients with a pCA < 5% were ultimately found to have benign disease. Although this approach avoids the potential harm of procedures, there is potential for interval growth of malignant nodules. The penalty for that growth is unknown and based on the biology of the tumor. One study of lung cancer experts found that there was little agreement in the penalty of missing growth between scans with estimates of stage shift from a lower stage to higher stage ranging from 1 to 50% with a resultant reduction in life expectancy.[16] On the opposite end of the spectrum, nodules with a pCA > 65% should be promptly resected in those healthy enough to tolerate surgery, providing both a diagnosis and treatment. The harms associated with this strategy include a morbidity of 5% and a mortality of 0.5%.[3]

Perhaps the most challenging group to manage are those with intermediate risk nodules (pCA 5%-65%), in which a substantial proportion of patients (45%) in this study are grouped and where 81% of the nodules were benign. Guidelines suggest further evaluation with PET scan, TTNA, or bronchoscopy.[3] Integrated PET/CT imaging has good sensitivity (86%- 91%) and specificity (71%- 81).[17] However, false-positive findings

CONFIDENTIAL AND PROPRIETARY INFORMATION

The information contained within this document may not be used, divulged, published, or otherwise disclosed without prior written consent of Biodesix.

(eg, granulomatous disease) and false-negative findings (eg, carcinoid) can mislead the clinician in either direction. TTNA has a yield between 70% (nodules < 15 mm) and 90% (nodules > 15 mm) but a 1% risk of hemorrhage and a pneumothorax rate of 16%, in which 6% require a chest tube.[3,18] The side-effect profile for bronchoscopy is acceptably low, but the yield (51%-70%) for pulmonary nodules even when using navigation is lower than TTNA.[19,20] In addition, a negative score from a bronchial-airway gene expression classifier of epithelial cells brushed from normal mucosa at the time of bronchoscopy improves the NPV in patients with an intermediate pretest probability for cancer and nondiagnostic bronchoscopy; however, not all patients with nodules routinely undergo bronchoscopy.[12] Clinicians can be left uncertain in management decisions, leading to potentially avoidable testing in patients with benign disease. One analysis found that of the total diagnostic costs of evaluating nodules, 43.1% was due to biopsy of patients without lung cancer.[21]

This intermediate-risk group may be those individuals in whom an integrated proteomics classifier would be useful. In patients with a pCA ≤ 50%, a likely benign test result could reduce the number of invasive procedures in those with benign nodules by 36% (an absolute risk reduction of 10.1% for all patients with benign nodules). This scenario assumes that all patients with likely benign results would be shifted to a surveillance strategy. It is important to consider that 3% of the patients with malignant nodules would also be placed in surveillance. Thus, it is critical for clinicians to continue to follow up until resolution or an acceptable period of CT radiographic stability has occurred.

What is unclear from these data is the extent to which clinicians would change their management based on the results of this test, although a survey of experienced pulmonologists found that a hypothetical blood test resulted in significant alterations in a decision to pursue invasive testing.[22] What is clear is that multiple studies reveal significant deviation from guideline-based care.[4,5,23] This trial is a clinical validation study that will need to undergo the clinical utility phase of biomarker development as outlined in the American Thoracic Society policy statement.[24]

The clinical validation phase of biomarker development establishes the accuracy of the biomarker in the intended-use population. Biomarker developers can use this accuracy, as well as an understanding of the potential benefit of a true result and the harm from a false result, to estimate whether the biomarker performs well enough to justify further evaluation in a clinical utility study. More than one method is available to help with this estimate.[24] One formula that has been proposed for a rule-out test states the specificity/(1 − sensitivity) ≥ (prevalence/1 − prevalence) × harm/benefit, where harm/benefit is the number of true negative results required to justify one false-negative result. If we use the prevalence of malignancy in the intended-use population in this study (16.3%) and the specificity (44%) and sensitivity (97%) reported here, the formula would produce a harm/benefit of 75.2 (ie, 75 true-negative results for each false-negative result). If the prevalence in the intended-use population was 40%, this number would be 22. These results strongly suggest that assessing the biomarker in a clinical utility study would be worthwhile.

Another important aspect of determining if the results of the clinical validation study warrant pursuit of a clinical utility study is understanding what percentage of tests ordered will provide a meaningful result. For a rule-out test, where the negative test result may change management but the positive test result will not, a test with the characteristics presented here could lead to management change in a population with a prevalence of 16.3% approximately 37% of the time, and in a population with a prevalence of 40%, approximately 28% of the time. This method provides less convincing evidence of the potential for clinical utility, but the implications for those with a negative test result are still believed to be large enough to support clinical utility testing. It is only through the results of a well-designed clinical utility study that we can determine the true potential value of the test.

The present study has limitations. First, the effect the plasma protein test might have had on test ordering was retrospectively analyzed; thus, a prospective study to assess changes in practice is warranted. Second, community practices are underrepresented in this trial; however, based on a previous study,[4] the likely benefit in terms of reduction of invasive testing would be significantly greater than that reported here. Third, although there is precedent for reporting 1-year outcomes for stable nodules, traditionally, 2 years of nodule stability is what has been required to determine a nodule is benign. Two-year follow-up data will be reported in the future once finalized. Fourth, there were 88 patients without follow-up CT scan data at 1 year. This degree of missing data may not be random, as patients with a low risk of lung cancer may have been

CONFIDENTIAL AND PROPRIETARY INFORMATION

The information contained within this document may not be used, divulged, published, or otherwise disclosed without prior written consent of Biodesix.

less likely to be adherent with follow-up recommendations.

This study has several strengths. First, to the best of our knowledge, this study is the largest prospective, multicenter, geographically diverse observational clinical study to validate an integrated blood biomarker for the evaluation of pulmonary nodules. Second, test development was rigorous: the integrated classifier was developed on an independent set of prospectively collected samples; the integrated classifier, laboratory standard operating procedures, and quality control subjects were all predefined and documented; and physicians, patients, and laboratory and statistical personnel were compliant with a blinding protocol. Finally, by having physicians estimate the probability of cancer in a pulmonary guideline, concordance with management decisions could be assessed.

Conclusions

This study is the first we are aware of to assess the accuracy of an integrated plasma proteomics classifier in patients with pulmonary nodules in a geographically diverse population with varying risk of cancer. In those with low to moderate risk nodules (pCA ≤ 50%), a "likely benign" test result could safely allow patients to be followed up by using serial imaging. Further research is needed to assess the effect of incorporating this test into the diagnostic algorithm for nodule management in the hope of reducing unnecessary procedures in patients without cancer.

Acknowledgments

Author contributions: P. K. had full access to all of the data in the study and takes responsibility for the integrity of and accuracy of the data analysis; K. C. F. and P. K. contributed substantially to the study design, data analysis and interpretation, and the writing of the manuscript; and G. A. S., N. T. T., A. V., P. P. M., A. P., S. C. S., D. M., and P. J. M. contributed substantially to data analysis and interpretation, and the writing of the manuscript.

Financial/nonfinancial disclosures: The authors have reported to *CHEST* the following: G. A. S. received research grant funding from Integrated Diagnostics, Exact Sciences, and Olympus. N. T. T. has received other grant funding from the American Cancer Society and the CHEST Foundation; industry grant funding from Exact Sciences, Veracyte, Integrated Diagnostics, Oncimmune, Olympus, and Cook Medical; consulting monies from Integrated Diagnostics, Olympus, Cook Medical, Veran, and Oncocyte; and has participated in advisory board meetings for Veracyte. P. K. is an employee and stockholder of Integrated Diagnostics. A. V. has received research funding from Integrated Diagnostics. A. P. is the Vice President of Clinical Operations and Medical Affairs for Integrated Diagnostics. S. C. S. is an employee of Integrated Diagnostics. K. C. F. was the Chief Medical Officer and stockholder at Integrated Diagnostics and authored on issued patents. D. M. received royalties from UpToDate for chapter authorship and editorship. P. J. M. has served on clinical advisory boards for Integrated Diagnostics, Oncimmune, Exact Sciences, Grail, and Nucleix; and has participated in speaking activities for Oncocyte.

*Writing Committee Members for the PANOPTIC Trial Study Team consisted of the following investigators and coordinators (the city/state or province in which their institution is located and the number of patients enrolled are shown in parentheses): G.A. Silvestri, MD, L. Leake (Charleston, SC; 78), P. Mazzone, MD, M. Beukemann (Cleveland, OH; 63), D. Midthun, MD, P. McCarthy (Rochester, MN; 55), B. Sigal, MD, T. Deluca (Winston-Salem, NC; 51), F. Laberge, MD, B. Fortin (Quebec City, QC, Canada; 47), M. Balaan, MD, B. Dimitt (Pittsburgh, PA; 46), A. Pierre, MD, F. Allison (Toronto, ON, Canada; 41), L. Yarmus, MD, K. Oakjones-Burgess (Baltimore, MD; 27), N. Tanner, MD, L. Leake (VA Medical Center, Charleston, SC; 23), N. Ettinger, MD, T. Setchfield (Chesterfield, MO; 23), D. Madtes, MD, J. Hubbard (Seattle, WA; 20), W. McConnell, MD, K. Robinson (Louisville, KY; 20), A. Lackey, MD, L. Jacques (Royal Oak, MI; 16), E. Kuo, MD, V. Markland-Gentles (Phoenix, AZ; 16), P. Massion, MD, A. Muterspaug, (Nashville, TN; 14), J. Leach, MD, K. Rothe (Minneapolis, MN; 14), W. Rom, MD, H. Pass, MD, A. Sorenson (New York, NY; 13), A. Chesnutt, MD, A. Georgeson (Portland, OR; 13), A. Balekian, MD, J. Fisher (Los Angeles, CA; 13), R. Murali, MD, A. Overton (Greensboro, NC; 12), N. Desai, MD, A. Levesque (Elk Grove, IL; 11), W. Krimsky, MD, S. King (Baltimore, MD; 9), A. Vachani, MD, K. Maletteri (Philadelphia, PA; 9), K. Mileham, MD, L. Carter (Charlotte, NC; 8), G. Hong, MD, J. Ma (San Francisco, CA; 8), K. Voelker, MD, H. Barrentine (Sarasota, FL; 7), R. Aronson, MD, M. Henderson (Tucson, AZ; 7), J. Lamberti, MD, C. Krawiecki (Falls Church, VA; 7), A. Case, MD, L. Wilkins (Austell, GA; 4), J. M. Ayers, MD, K. Fangmann (Danville, PA; 4), J. Landis, MD, L. DeSouza (Springfield, MA; 3), Z. Hammoud, MD, D. Kah (Detroit, MI; 3), and J. Sanchez, MD, L. Murdoch, (Temple, TX; 0).

Role of sponsors: Integrated Diagnostics designed the trial, collected the data, and assisted in interpretation of the results.

Additional information: The e-Figures and e-Tables can be found in the Supplemental Materials section of the online article.

References

1. Gould MK, Tang T, Liu IL, et al. Recent trends in the identification of incidental pulmonary nodules. *Am J Respir Crit Care Med.* 2015;192(10):1208-1214.
2. National Lung Screening Trial Research Trial, Aberle DR, Adams AM, et al. Reduced lung-cancer mortality with low-dose computed tomographic screening. *N Engl J Med.* 2011;365(5):395-409.
3. Gould MK, Donington J, Lynch WR, et al. Evaluation of individuals with pulmonary nodules: when is it lung cancer? Diagnosis and management of lung cancer, 3rd ed: American College of Chest Physicians evidence-based clinical practice guidelines. *Chest.* 2013;143(suppl 5):e93S-120S.
4. Tanner NT, Aggarwal J, Gould MK, et al. Management of pulmonary nodules by community pulmonologists: a multicenter observational study. *Chest.* 2015;148(6):1405-1414.
5. Tanner NT, Porter A, Gould MK, Li XJ, Vachani A, Silvestri GA. Physician assessment of pretest probability of malignancy and adherence with guidelines for pulmonary nodule evaluation. *Chest.* 2017;152(2):263-270.
6. Li XJ, Hayward C, Fong PY, et al. A blood-based proteomic classifier for the molecular characterization of pulmonary nodules. *Sci Translation Med.* 2013;5(207):207ra142.
7. Vachani A, Hammoud Z, Springmeyer S, et al. Clinical utility of a plasma protein classifier for indeterminate lung nodules. *Lung.* 2015;193(6):1023-1027.
8. Grassadonia A, Tinari N, Iurisci I, et al. 90K (Mac-2 BP) and galectins in tumor progression and metastasis. *Glycoconj J.* 2002;19(7-9):551-556.

CONFIDENTIAL AND PROPRIETARY INFORMATION

The information contained within this document may not be used, divulged, published, or otherwise disclosed without prior written consent of Biodesix.

ARTICLE IN PRESS

9. Yang L, Wang F, Wang L, et al. CD163+ tumor-associated macrophage is a prognostic biomarker and is associated with therapeutic effect on malignant pleural effusion of lung cancer patients. *Oncotarget*. 2015;6(12):10592-10603.

10. Moestrup SK, Moller HJ. CD163: a regulated hemoglobin scavenger receptor with a role in the anti-inflammatory response. *Ann Med*. 2004;36(5):347-354.

11. Kearney P, Hunsucker SW, Li XJ, Porter A, Springmeyer S, Mazzone P. An integrated risk predictor for pulmonary nodules. *PLoS One*. 2017;12(5):e0177635.

12. Silvestri GA, Vachani A, Whitney D, et al. A bronchial genomic classifier for the diagnostic evaluation of lung cancer. *N Engl J Med*. 2015;373(3):243-251.

13. Fagerland MW, Lydersen S, Laake P. The McNemar test for binary matched-pairs data: mid-p and asymptotic are better than exact conditional. *BMC Med Res Methodol*. 2013;13:91.

14. Swensen SJ, Silverstein MD, Ilstrup DM, Schleck CD, Edell ES. The probability of malignancy in solitary pulmonary nodules. Application to small radiologically indeterminate nodules. *Arch Intern Med*. 1997;157(8):849-855.

15. Gould MK, Ananth L, Barnett PG; Veterans Affairs SNAP Cooperative Study Group. A clinical model to estimate the pretest probability of lung cancer in patients with solitary pulmonary nodules. *Chest*. 2007;131(2):383-388.

16. Schultz EM, Silvestri GA, Gould MK. Variation in experts' beliefs about lung cancer growth, progression, and prognosis. *J Thorac Oncol*. 2008;3(4):422-426.

17. Deppen SA, Blume JD, Kensinger CD, et al. Accuracy of FDG-PET to diagnose lung cancer in areas with infectious lung disease: a meta-analysis. *JAMA*. 2014;312(12):1227-1236.

18. Wiener RS, Wiener DC, Gould MK. Risks of transthoracic needle biopsy: how high? *Clin Pulm Med*. 2013;20(1):29-35.

19. Ost DE, Ernst A, Lei X, et al. Diagnostic yield and complications of bronchoscopy for peripheral lung lesions. Results of the AQuIRE registry. *Am J Respir Critic Care Med*. 2016;193(1):68-77.

20. Wang Memoli JS, Nietert PJ, Silvestri GA. Meta-analysis of guided bronchoscopy for the evaluation of the pulmonary nodule. *Chest*. 2012;142(2):385-393.

21. Lokhandwala T, Bittoni MA, Dann RA, et al. Costs of diagnostic assessment for lung cancer: a Medicare claims analysis. *Clin Lung Cancer*. 2017;18(1):e27-e34.

22. Vachani A, Tanner NT, Aggarwal J, et al. Factors that influence physician decision making for indeterminate pulmonary nodules. *Ann Am Thorac Soc*. 2014;11(10):1586-1591.

23. Wiener RS, Gould MK, Slatore CG, Fincke BG, Schwartz LM, Woloshin S. Resource use and guideline concordance in evaluation of pulmonary nodules for cancer: too much and too little care. *JAMA Intern Med*. 2014;174(6):871-880.

24. Mazzone PJ, Sears CR, Arenberg DA, et al. Evaluating molecular biomarkers for the early detection of lung cancer: when is a biomarker ready for clinical use? An official American Thoracic Society policy statement. *Am J Respir Crit Care Med*. 2017;196(7):e15-e29.

CONFIDENTIAL AND PROPRIETARY INFORMATION

The information contained within this document may not be used, divulged, published, or otherwise disclosed without prior written consent of Biodesix.
